# Beclin 1 Is Required for Neuron Viability and Regulates Endosome Pathways via the UVRAG-VPS34 Complex

**DOI:** 10.1371/journal.pgen.1004626

**Published:** 2014-10-02

**Authors:** Nicole C. McKnight, Yun Zhong, Mitchell S. Wold, Shiaoching Gong, Greg R. Phillips, Zhixun Dou, Yanxiang Zhao, Nathaniel Heintz, Wei-Xing Zong, Zhenyu Yue

**Affiliations:** 1 Department of Neurology and Neuroscience, Friedman Brain Institute, Icahn School of Medicine at Mount Sinai, New York, New York, United States of America; 2 Laboratory of Molecular Biology, Howard Hughes Medical Institute, Rockefeller University, New York, New York, United States of America; 3 Department of Molecular Genetics and Microbiology, Stony Brook University, Stony Brook, New York, United States of America; 4 Department of Applied Biology and Chemical Technology, State Key Laboratory of Chirosciences, The Hong Kong Polytechnic University, Hung Hom, Kowloon, Hong Kong, China; University of Minnesota, United States of America

## Abstract

Deficiency of autophagy protein beclin 1 is implicated in tumorigenesis and neurodegenerative diseases, but the molecular mechanism remains elusive. Previous studies showed that Beclin 1 coordinates the assembly of multiple VPS34 complexes whose distinct phosphatidylinositol 3-kinase III (PI3K-III) lipid kinase activities regulate autophagy at different steps. Recent evidence suggests a function of beclin 1 in regulating multiple VPS34-mediated trafficking pathways beyond autophagy; however, the precise role of beclin 1 in autophagy-independent cellular functions remains poorly understood. Herein we report that beclin 1 regulates endocytosis, in addition to autophagy, and is required for neuron viability *in vivo*. We find that neuronal beclin 1 associates with endosomes and regulates EEA1/early endosome localization and late endosome formation. Beclin 1 maintains proper cellular phosphatidylinositol 3-phosphate (PI(3)P) distribution and total levels, and loss of beclin 1 causes a disruption of active Rab5 GTPase-associated endosome formation and impairment of endosome maturation, likely due to a failure of Rab5 to recruit VPS34. Furthermore, we find that Beclin 1 deficiency causes complete loss of the UVRAG-VPS34 complex and associated lipid kinase activity. Interestingly, beclin 1 deficiency impairs p40^phox^-linked endosome formation, which is rescued by overexpressed UVRAG or beclin 1, but not by a coiled-coil domain-truncated beclin 1 (a UVRAG-binding mutant), Atg14L or RUBICON. Thus, our study reveals the essential role for beclin 1 in neuron survival involving multiple membrane trafficking pathways including endocytosis and autophagy, and suggests that the UVRAG-beclin 1 interaction underlies beclin 1's function in endocytosis.

## Author Summary

Beclin 1 was not only the first-described mammalian autophagy protein, but is one of the most widely-characterized players in autophagy regulation. It is implicated in multiple human disease conditions. As a core component of the essential lipid kinase complex (PI3K-III), beclin 1 has largely been characterized to date in the context of autophagy through its recruitment of additional autophagy proteins for the assembly of the PI3K-III complexes involved in the nucleation of the autophagosome. Little is known, however, about how beclin 1 regulates specific functions of PI3K-III in other membrane trafficking pathways. Furthermore, although beclin 1 has been linked to multiple neurodegenerative diseases, the function of beclin 1 in the brain remains uncharacterized. Herein, we used genetic animal models and mutant cell lines to demonstrate that beclin 1 participates in multiple organelle trafficking pathways. Beclin 1 deficiency in neurons causes severe neurodegeneration, concomitant with aberrant late endosome formation and impaired phospholipid localization. Our mechanistic study reveals the essential role for beclin 1 in neuron survival involving multiple membrane trafficking pathways including endocytosis and autophagy. Our study clarifies the physiological function of beclin 1, which leads for further understanding of its role in tumorigenesis, infectious disease and neurodegenerative disease.

## Introduction

Autophagy is a regulated “self-digestion” pathway in the cytoplasm of cells involving the synthesis, trafficking and delivery of autophagosomes to lysosomes for degradation. Beclin 1 was the first described mammalian autophagy protein and a core component of the class III phosphatidylinositol 3-kinase (PI3K-III) complex, which plays an important role in membrane trafficking and restructuring involved in autophagy, endocytosis, cytokinesis and phagocytosis [1]. To date beclin 1 has largely been characterized in the context of autophagy; it modulates the lipid kinase activity of PI3K-III catalytic unit VPS34, which generates phosphatidylinositol 3-phosphate (PI(3)P), enabling the recruitment of the other autophagy proteins involved in the nucleation of autophagosome [2], [3]. Little is known, however, about how beclin 1 regulates specific functions of VPS34. Multiple lines of evidence have linked altered expression of beclin 1 to several major human diseases such as cancer, infectious diseases and neurodegenerative disorders [4], [5]. It is thus imperative to understand the precise molecular mechanism whereby beclin 1 regulates VPS34 functions in autophagy and other membrane trafficking pathways, the disruption of which may underlie the pathogenesis of multiple diseases.

Our previous report and also others demonstrated that mammalian beclin 1-VPS34 forms multiple complexes [6]–[8]. The three core proteins beclin 1, VPS34 and VPS15, interact with a number of auxiliary proteins such as Atg14L, UVRAG, RUBICON, Ambra1 and Bif-1. By recruiting different binding partners and forming distinct PI3K-III complexes, beclin 1 modulates PI3K-III activity and regulates autophagy at multiple stages including nucleation and maturation. Moreover, beclin 1 is modified post-translationally by phosphorylation and ubiquitination serving to fine-tune PI3K-III activity and autophagy in response to various intracellular and extracellular signals [9].

Early reports implicate beclin 1 in numerous neuropathological conditions [10], [11] and emerging evidence demonstrates the neuroprotective function of beclin 1 through removal of disease-related proteins including APP metabolites [12], α-synuclein [13] and mutant ataxin [14] in the nervous system. However, the physiological function of beclin 1 in neurons is poorly understood and whether the neuroprotective function of beclin 1 is mediated through strictly autophagy-dependent or independent pathways is unclear. Here we report that disruption of beclin 1 in a neuron-type specific manner results in severe neurodegeneration in mice. Neuronal beclin 1 is associated with endocytic compartments and ablation of *Becn1* causes impairment of the endosome pathway. Our results show a role for beclin 1 in regulating PI3K-III activity and endocytosis via the beclin 1-UVRAG interaction, highlighting beclin 1's physiological function in multiple membrane traffic pathways.

## Results

### Accelerated neurodegeneration in *Becn1* conditional knockout mice

**Figure 1:**
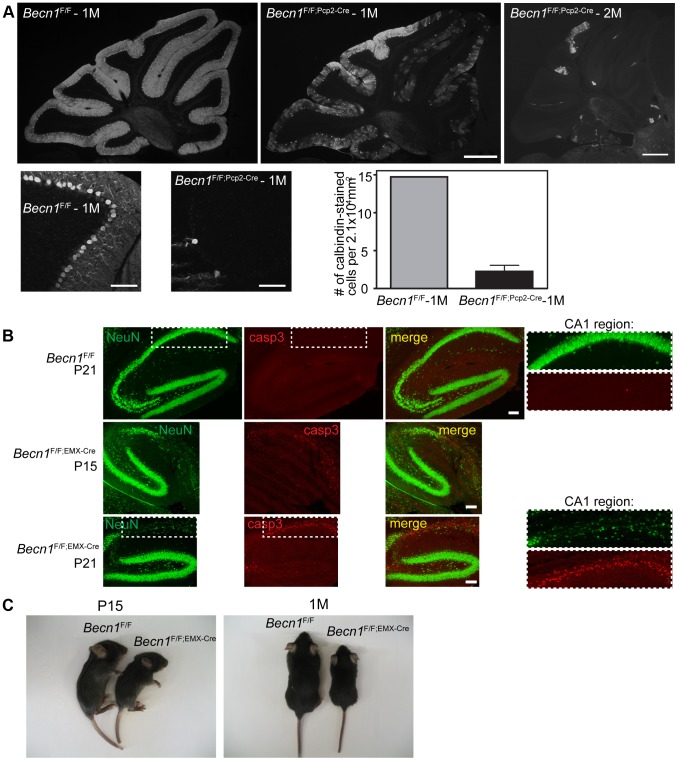
Loss of beclin 1 in selected neurons leads to rapid degeneration. **A**. Severe PC loss is observed at 1 month (1M) and 2 months (2M) in *Becn1* cKO mice. Calbindin 1 staining of cerebellum PCs from (left to right) *Becn1* ^F/F^ 1M, *Becn1* ^F/F;Pcp2-Cre^ 1M and *Becn1* ^F/F;Pcp2-Cre^ 2M mice. Scale bar = 0.5 mm. Lower panels show 20× images of *Becn1* ^F/F^ and *Becn1* ^F/F;Pcp2-Cre^ cerebellums at 1M. Scale bars = 100 µm. Quantification of 11 images each for 1 control and 3 cKO mice; bars are means +/− s.e.m. (n = 3). **B**. cKO of *Becn1* in the hippocampus/cortex leads to caspase 3-dependent cell death. NeuN and caspase3 staining of *Becn1* ^F/F^ and *Becn1* ^F/F;EMX-Cre^ hippocampi from mice at indicated ages. Scale bars = 100 µm. Zoomed boxes show CA1 region. **C**. cKO of *Becn1* in the hippocampus/cortex leads to reduced body size. *Becn1* ^F/F;EMX-Cre^ hippocampus/cortex-specific KO mice show reduced size. Photos of *Becn1* ^F/F^ and *Becn1* ^F/F;EMX-Cre^ mice at P15, 1M.

Our previous reports showed that *Becn1* deletion causes embryonic lethality at E7.5, preventing us from studying beclin 1 physiological functions in adult tissues [15]. We generated a conditional knock-out (cKO) mouse line by crossing *Becn1* Flox/Flox (*Becn1* ^F/F^) mice with Pcp2-Cre transgenic mice [16] to delete *Becn1* specifically in cerebellar Purkinje cells (PCs) (Figure S1A, S1B). In *Becn1* ^F/F;Pcp2-Cre^ mice, a loss of beclin 1 expression is observed in cerebellar PCs at P21 (Figure S1C). In contrast to *Becn1* ^F/F^ control mice, *Becn1* ^F/F;Pcp2-Cre^ cKO mice developed rapid degeneration of PCs between P21 (Figure S1D) and 1 month (>80% loss) and lost nearly all PCs at 2 months (Figure 1A). The loss of PCs is associated with abnormal gait and ataxic behavior (Figure S2A, Movie S1, S2). Thus, *Becn1* cKO mice have clearly accelerated PC degeneration, differing significantly from autophagy gene *Atg7* conditional knockout (*Atg7* ^F/F;Pcp2-Cre^ cKO) mice, in which PCs are intact at 1 month and only a moderate percentage of PCs degenerated by 2 months, as we previously reported [16]. The relatively late onset of PC degeneration in *Atg7* cKO mice is unlikely due to delayed deletion of the *Atg7* gene because Atg7 cKO mice were derived by crossing *Atg7* ^F/F^ to the same Pcp2-Cre transgenic mice used for *Becn1* cKO and loss of Atg7 protein is apparent at P19 [16], at a similar stage as seen in *Becn1* ^F/F;Pcp2-Cre^ mice (Figure S1C). The enhanced neuronal loss in *Becn1* conditional KO mice is consistent with the early embryonic lethality of conventional *Becn1* KO mice [15] as opposed to the neonatal death of conventional *Atg7* KO mice [17].

To evaluate the impact of beclin 1 deletion on the viability of neurons, we generated a second *Becn1* cKO mouse line *Becn1* ^F/F;EMX-Cre^ by crossing *Becn1* ^F/F^ with EMX-Cre transgenic mice to delete *Becn1* in cortical and hippocampal neurons [18]. Ablation of *Becn1* in hippocampal neurons indeed caused even earlier and more severe neurodegeneration than mutant PCs as shown by the overt reduction of neuron density at the CA1 region of the hippocampus in *Becn1* ^F/F;EMX-Cre^ mice at postnatal day 15 and 21 (Figure 1B). The degeneration of CA1 neurons was clearly associated with activated caspase 3 activity, suggesting that lack of beclin 1 leads to apoptotic neuron death (Figure 1B). In contrast, a recent study reported that deletion of Atg7 causes no loss of CA1 neurons at 3 months and only modest reduction of CA1 neurons at 15 months [19]. Consistently, we showed that lack of *Atg7* causes slow, protracted dopamine neuron loss starting as late as 9 months [20]. Compared to *Becn1* ^F/F^ mice, *Becn1* ^F/F;EMX-Cre^ mice showed reduced body size from as early as P15, which persisted throughout postnatal development (Figure 1C; Figure S2B). We observed mortality in *Becn1* ^F/F;EMX-Cre^ mice from the age of 6 weeks, and almost all mutant mice died by the age of 4 months. The phenotype of *Becn1* ^F/F;EMX-Cre^ mice again demonstrated the significant enhancement of neurotoxicity caused by loss of beclin 1 compared to Atg7, suggesting that beclin 1 is associated with cell survival mechanisms distinct from Atg7-mediated autophagy.

We found swollen dystrophic axons of the PCs projected to the deep cerebellar nuclei (DCN) in *Becn1* ^F/F;Pcp2-Cre^ mice (Figure S2C). This observation is consistent with our study of *Atg7* ^F/F;Pcp2-Cre^ mice in which autophagy disruption causes axon dystrophy containing aberrant membrane structures [16]. In addition, *Becn1* ^F/F;EMX-Cre^ mice showed a clear increase in p62 levels in hippocampal neurons and cortical neurons compared to the control (Figure S3A). Immunofluorescence staining confirmed the enhancement of p62 levels in the cytoplasm of the CA1 region of *Becn1* cKO mice (Figure S3B), whereas p62 staining was detected at background levels in control mice, consistent with the Westernblot analysis (Figure S3A). We expressed transgene GFP-LC3 in *Becn1* ^F/F;EMX-Cre^ mice and immunofluorescence staining showed that GFP-LC3 accumulated in the CA1 region together with elevated p62 staining (Figure S3C); in contrast, little GFP-LC3 signal was seen in the CA1 region of control *Becn1* ^F/F^ mice. Ubiquitinated protein levels were also increased in mutant CA1 areas compared to control (Figure S3D). Thus, as expected, lack of beclin 1 also caused impaired autophagy-lysosomal degradation in neurons.

### Beclin 1 is associated with endosomes in neurons

**Figure 2:**
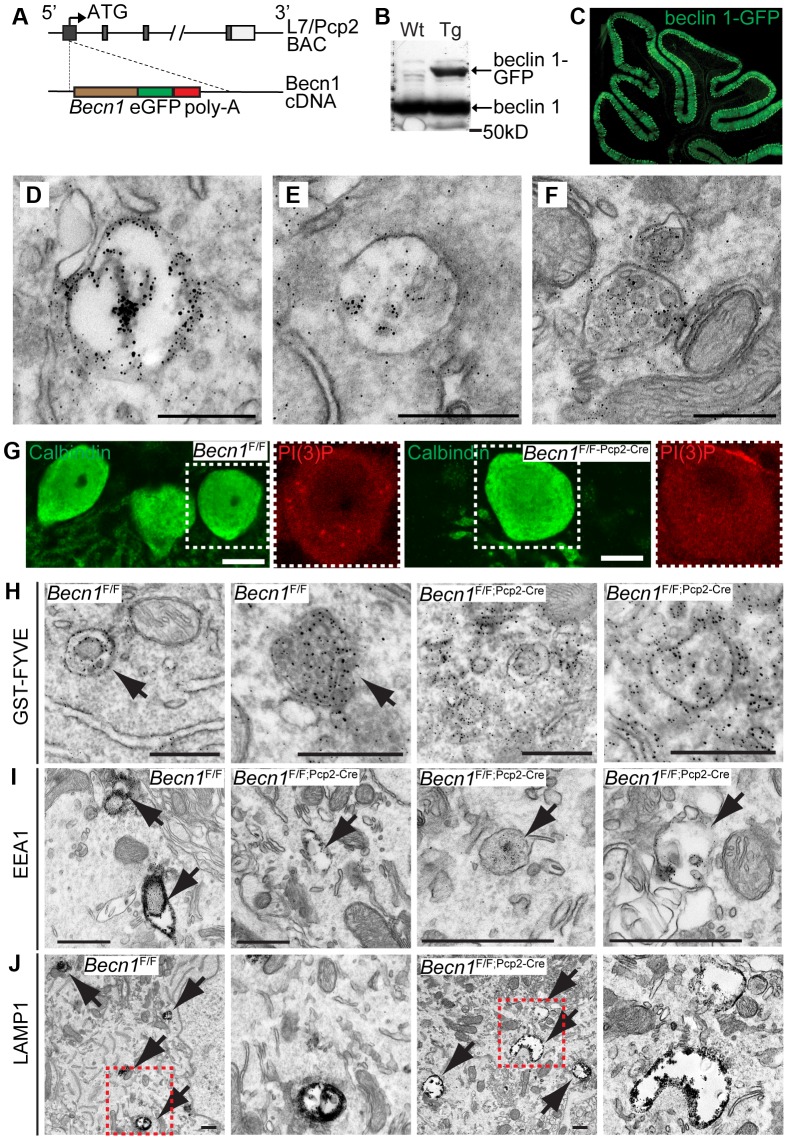
beclin 1-GFP expressed in the Purkinje cells of transgenic mice associates with endosomal organelles. **A**. L7-*Becn1*-eGFP mice were generated using bacterial artificial chromosome (BAC) recombination techniques [52]. Schematic of the *Becn1* gene fused to eGFP used to produce beclin 1-GFP in PCs using the L7/Pcp2 gene on BAC. **B**. beclin 1-GFP and endogenous beclin 1 are expressed in the brain. Anti-beclin 1 blot shows beclin 1-GFP in transgenic (Tg) mice. **C**. Transgenically expressed beclin 1-GFP is visualized specifically in the PCs of the cerebellum with anti-GFP antibody. Fluorescent image of the cerebellum of an L7-*Becn1*-GFP transgenic mouse. **D**.–**F**. beclin 1-GFP is found on membrane structures in PCs. Immuno-EM of the cerebellum of P21 L7-*Becn1*-GFP transgenic mice showing beclin 1-GFP on endosomes (**D**) and MVBs (**E**),(**F**). Scale bars = 250 nm. Loss of beclin 1 in Purkinje cells causes mislocalization of PI(3)P and abnormal endosomes and endolysosomes. **G**. Endogenous PI(3)P is punctate in *Becn1* ^F/F^ PCs but diffuse in *Becn1* ^F/F-Pcp2-Cre^ PCs. Immunofluorescent images of calbindin and PI(3)P stained *Becn1* ^F/F^ and *Becn1* ^F/F;Pcp2-Cre^ cerebellums of 1M mice. Dashed white boxes indicate zoomed image. Scale bars = 10 µm. **H**. GST-FYVE localizes to membranes in *Becn1* ^F/F^ PCs but is diffuse in *Becn1* ^F/F-Pcp2-Cre^ PCs. Immuno-EM images of cerebellar slices incubated with GST-FYVE and probed with anti-GST antibody. Scale bars = 200 nm. Arrow indicates endosome. **I**. EEA1 staining is intense in early endosomal structures in *Becn1* ^F/F^ control PCs but is reduced significantly on endosomes in *Becn1* ^F/F-Pcp2-Cre^ PCs. Immuno-EM micrographs of P21 *Becn1* ^F/F^ or *Becn1* ^F/F-Pcp2-Cre^ PCs stained with EEA1. Scale bars = 500 nm. **J**. LAMP1-stained vesicles are abnormally shaped and expanded in *Becn1* ^F/F-Pcp2-Cre^ cKO PCs. Immuno-EM micrographs of P21 *Becn1* ^F/F^ or *Becn1* ^F/F-Pcp2-Cre^ cKO PCs stained with LAMP1. Dashed red boxes indicate zoomed image. Scalebars = 500 nm.

To understand the cellular function of beclin 1 in neurons, we examined our newly-established BAC transgenic mouse line expressing beclin 1-GFP (green fluorescent protein) fusion protein in Purkinje cells under the control of the Pcp2 promoter (Figure 2A). Because of the poor quality of available anti-beclin 1 antibodies, our ability to detect endogenous beclin 1 localization was limited. Using a highly specific GFP antibody, we successfully detected GFP-LC3 subcellular localization in neurons assisted by immuno-EM analysis [21]. We therefore used the same anti-GFP antibody to examine beclin 1-GFP subcellular localization in neurons. Pcp2-BAC transgenics directed beclin 1-GFP expression selectively in cerebellar PCs (Figure 2B,C). We performed immuno-EM with anti-GFP antibody and found that immuno-reactive signal is enriched in endosomes suggesting that beclin 1-GFP is localized at endosomes (Figure 2D,E,F). This observation is in contrast to GFP-LC3 signals, which specifically labeled autophagosomes in the dystrophic axon terminals of PCs as reported by us previously [21].

### Loss of beclin 1 causes PI(3)P mislocalization in neurons and abnormal endosome formation in *Becn1* null neurons

We next examined PI(3)P localization in PCs of *Becn1* ^F/F^ and *Becn1* ^F/F;Pcp2-Cre^ mice by immunofluorescence and found that while control PCs contain PI(3)P puncta, the PI(3)P signal was diffuse throughout *Becn1* cKO cells (Figure 2G) suggesting that without beclin 1, PI(3)P was not appropriately localized. We next performed immuno-EM to confirm the observed PI(3)P localization using a GST-FYVE domain fusion protein that specifically binds to endogenous PI(3)P [22], [23]. In control brains GST-FYVE labeled endosomes, while in *Becn1* cKO PCs GST-FYVE was not enriched in specific membrane structures and instead appeared diffuse throughout the cytosol (Figure 2H). We therefore conclude that endogenous beclin 1 is critical for proper targeting of PI(3)P to multiple membrane vesicles (e.g. endosomes) and thus the maintenance of the membrane homeostasis in neurons.

The localization of beclin 1-GFP on endosomes (Figure 2 D,E,F) and impaired PI(3)P localization in beclin 1-deficient neurons (Figure 2 G,H) implicates a function for beclin 1 in the endocytic pathway. We performed immuno-EM to examine endosomes in PCs using antibodies against the early endosome marker EEA1 and the late endosome/lysosome marker LAMP1. In control *Becn1* ^F/F^ PCs, EEA1 antibody-stained endosomes showed much stronger intensity (indicated by dense dark signals) than those in *Becn1* ^F/F;Pcp2-Cre^ PCs (Figure 2I), suggesting impaired recruitment of EEA1 to endosomes in the absence of beclin 1. Next we observed that LAMP1-labeled vacuoles in *Becn1* ^F/F;Pcp2-Cre^ PCs were significantly enlarged compared to those in control *Becn1* ^F/F^ PCs, suggesting aberrant formation of late endosome/lysosome in the absence of beclin 1 (Figure 2J).

### *Becn1* deficiency is associated with impaired endosome maturation and EGFR degradation

**Figure 3:**
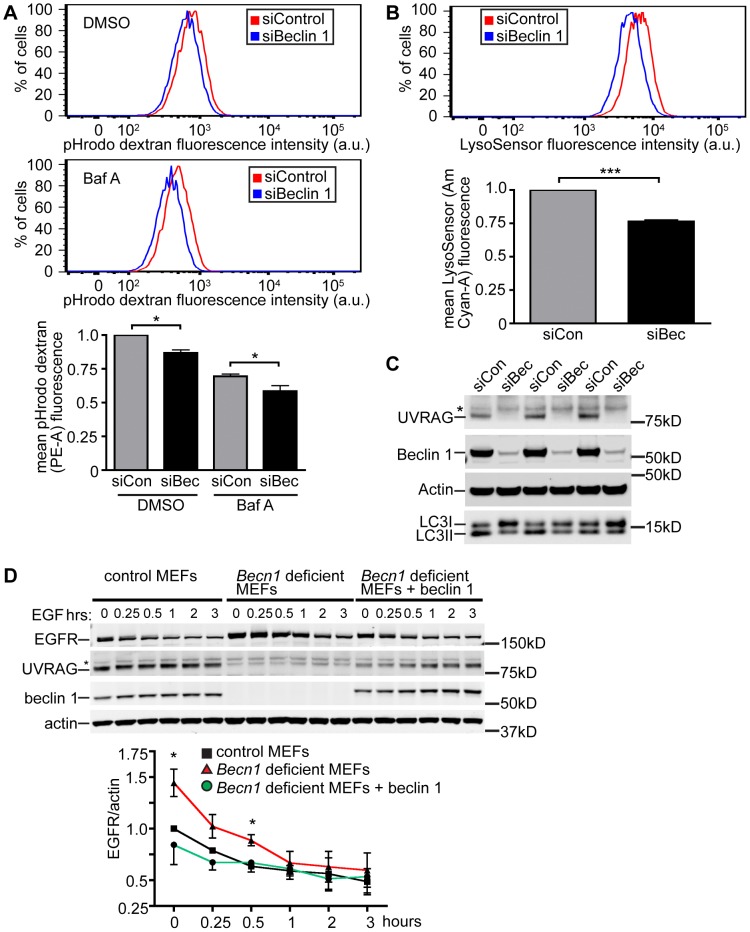
Beclin 1 deficient HeLa cells and MEFs display decreased endocytosis. **A**. Hela cells treated with indicated siRNA (siCon is siControl; siBec is siBeclin 1) were treated with DMSO or Bafilomycin A1 (BafA). Representative composite fluorescence plots and normalized quantification of mean fluorescence intensity of 10,000 cells were determined from three separate experiments. Mean pHrodo dextran (PE-A) fluorescence; bars represent mean +/− s.e.m. (n = 3). DMSO pHrodo dextran siCon vs. siBec one sample t-test (two-tailed) p = 0.0283; BafA pHrodo dextran siCon vs. siBec one-tailed t-test p = 0.0246. **B**. Mean LysoSensor (Am Cyan-A) fluorescence intensity; bars represent mean +/− s.e.m. (n = 3). LysoSensor siCon vs. siBec one sample t-test (two-tailed) p = 0.0029. **C**. Anti-UVRAG, -Beclin 1, -Actin and –LC3 blots of HeLa cells lysed after indicated siRNA knock-down. Asterix indicates unspecific band. **D**. Receptor-mediated endocytosis as measured by EGFR internalization is inhibited in *Becn1* deficient MEFs and rescued with re-introduction of beclin 1. Anti-EGFR, - UVRAG, -beclin 1 and –actin blots of control or *Becn1* deficient MEFs lysed after indicated treatment with EGF. Asterix indicates unspecific band. Quantification of normalized EGFR/actin from 3 separate experiments. Bars represent mean +/− s.e.m. (n = 3); p = 0.0395, 0.0208 (one-tailed t-test).

A previous study reported impaired internalization of epidermal growth factor receptor (EGFR), a process related to endocytosis, after Beclin 1 knock-down in HeLa cells [24], implicating a role for beclin 1 in the endocytic pathway. To validate the effect of beclin knock-down in the endocytic pathway we examined the uptake of pHrodo Red dextran that occurs during endocytosis associated with endosomal acidification [25]. We performed flow cytometry and observed that the mean fluorescence intensity in Beclin 1 deficient HeLa cells incubated with pHrodo dextran was lower than that of control cells, consistent with reduced transport to increasingly acidic endosomes (Figure 3A). Cells treated with the endosomal acidification inhibitor bafilomycin A showed overall reduced fluorescence by flow cytometry, which was further inhibited after Beclin 1 knock-down. Furthermore, Beclin 1 deficient cells showed decreased mean fluorescence of LysoSensor green dye suggesting an inhibition of endosome maturation (Figure 3B) [26]. We noticed that Beclin 1 knock-down in HeLa cells caused altered LC3II/LC3I ratios and near abolishment of UVRAG protein levels (Figure 3C). The result provides support to the notion that beclin 1 plays a role in late endosome formation (Figure 2J) or endosome maturation, in addition to autophagy.

To assist the characterization of beclin 1 in the endocytic pathway, we generated genetic *Becn1* deficient murine embryonic fibroblasts (MEFs). We next examined the impact of loss of beclin 1 in cells' ability to internalize and degrade EGFR upon EGF stimulation. We observed significantly increased EGFR protein levels in *Becn1* deficient MEFs at steady state and early times points (0.5 h) post EGF treatment compared to control MEFs suggesting that EGFR is accumulated and not properly degraded without beclin 1 (Figure 3D). The defect was rescued by ectopic and stable expression of beclin 1 in *Becn1* deficient MEFs (‘revertant MEFs’).

### Reduced PI(3)P levels and VPS34 activity in *Becn1* deficient cells

**Figure 4:**
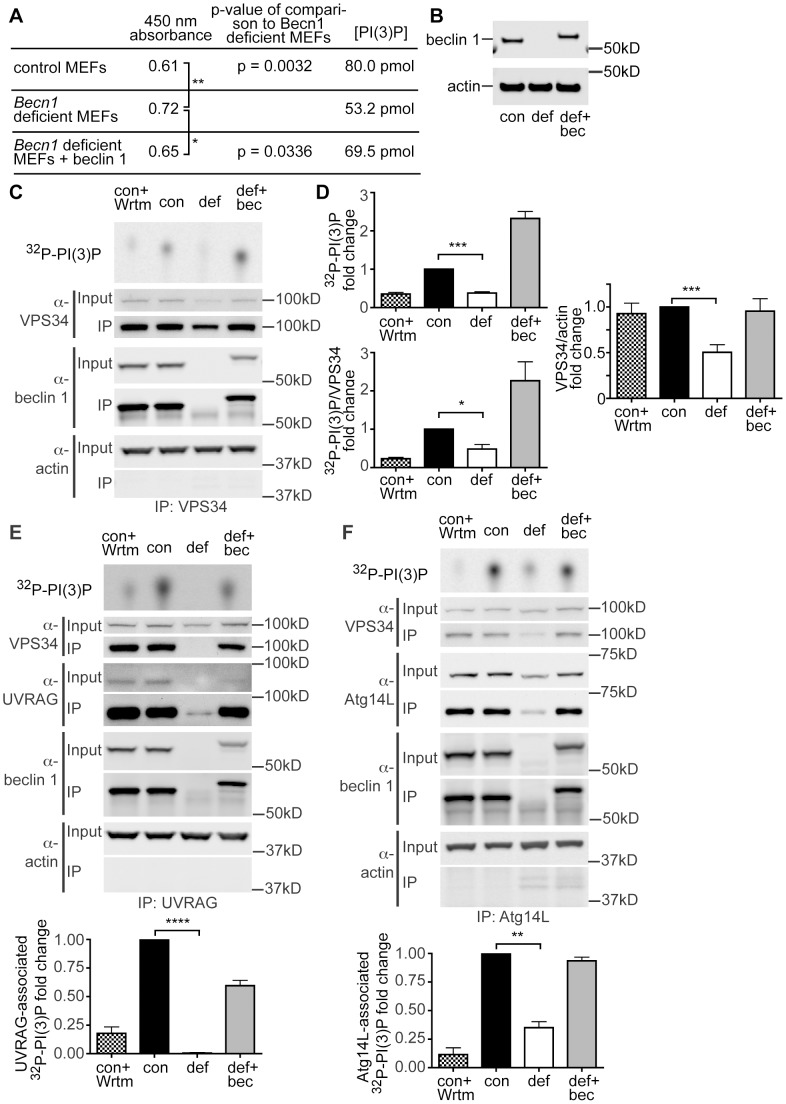
PI(3)P and VPS34 lipid kinase activity is decreased in *Becn1* deficient MEFs. **A**. Table showing results from PI(3)P ELISA. Average 450 nm absorbance, p-value of comparison to *Becn1* deficient MEFs. PI(3)P concentration is determined by comparison to a standard curve. Quantification of 450 nm absorbance of control, *Becn1* deficient, and *Becn1* revertant MEFs (6,5,6 replicates); statistics using a one-tailed t-test p = 0.0032, 0.0336. **B**. Anti-beclin 1, and -actin blots of MEF cell lysates. Con is control MEFs, def is *Becn1* deficient MEFs, and rev is *Becn1* revertant MEFs plus beclin 1. **C**. Autoradiograph of ^32^P-labelled PI(3)P and anti-VPS34, -beclin 1, and -actin blots after VPS34 IP of lysates from indicated MEFs with or without wortmannin (Wrtm) treatment. **D**. Quantification of normalized ^32^P-labelled PI(3)P and ^32^P-PI(3)P/VPS34 (from IP) from 3 separate experiments and VPS34/actin (from input lanes) from 9 separate experiments (including Fig. 4 E, F). Con is control MEFs, def is *Becn1* deficient MEFs and def+bec is *Becn1* revertant MEFs. Bars represent mean +/− s.e.m. (n = 3) con vs. def p = 0.0003, p = 0.0226, and p = 0.0002 top to bottom using a one sample t-test (two-tailed). UVRAG and Atg14-associated PI(3)P production is decreased in *Becn1* deficient MEFs. **E**. Autoradiograph of ^32^P-labelled PI(3)P and anti-VPS34, -UVRAG, -beclin 1, and -actin blots after UVRAG IP of lysates from indicated MEFs with or without wortmannin (Wrtm) treatment. Con is control MEFs, def is *Becn1* deficient MEFs and def+bec is *Becn1* revertant MEFs. Quantification of normalized ^32^P-labelled PI(3)P from 3 separate experiments. Bars represent mean +/− s.e.m. (n = 3) con vs. def p<0.0001 using a one sample t-test (two-tailed). **F**. Autoradiograph of ^32^P-PI(3)P and anti-VPS34, -Atg14L, -beclin 1, and -actin blots after Atg14L IP of lysates from indicated MEFs with or without wortmannin (Wrtm) treatment. Quantification of normalized ^32^P-labelled PI(3)P from 3 separate experiments. Bars represent mean +/− s.e.m. (n = 3) con vs. def p = 0.0065 using a one sample t-test (two-tailed).

Our study of beclin 1 deficient neurons showed an aberrant distribution of PI(3)P (Figure 2G,H). It is unclear, however, whether beclin 1 deficiency affects intracellular PI(3)P and VPS34 protein levels. We next performed an ELISA for competitive binding to a PI(3)P-coated plate to determine the mass amount of PI(3)P cells in *Becn1* deficient MEFs. The colorimetric signal was read at absorbance 450 nm and is inversely proportional to the amount of PI(3)P extracted from cells. The result indicates that intracellular PI(3)P content was significantly reduced in *Becn1* deficient MEFs which was rescued in revertant MEFs (Figure 4A,B).

We also examined VPS34 protein levels and VPS34-associated lipid kinase activity in *Becn1* deficient MEFs. Control MEFs, *Becn1* deficient MEFs, and *Becn1* revertant MEFs were immunoprecipitated with VPS34 antibody and ^32^P-labelled PI(3)P was determined using thin-layer chromatography (TLC). As expected, control MEFs treated with the lipid kinase inhibitor wortmannin showed decreased ^32^P-PI(3)P (Figure 4C,D). *Becn1* deficient MEFs showed decreased total ^32^P-PI(3)P levels and levels of ^32^P-PI(3)P normalized to the amount of VPS34 immunoprecipitated. The ^32^P-PI(3)P deficit was rescued with reintroduction of beclin 1 in the revertant MEFs. In addition, protein levels of VPS34 were also decreased in *Becn1* deficient MEFs (Figure 4C,D). These results suggests that reduced levels of ^32^P-PI(3)P in *Becn1* deficient MEFs is likely due to both decreased VPS34 protein levels and decreased VPS34 lipid kinase activity. Thus our results show that loss of beclin 1 caused a reduction of VPS34 protein level in addition to a decrease in VPS34 lipid kinase activity, leading to a decrease of production of intracellular PI(3)P, which in turn leads to an impairment of endocytosis in beclin 1 deficient cells and neurons.

### Lack of beclin 1 results in abolishment of the UVRAG-VPS34 complex and UVRAG-associated VPS34 activity

UVRAG and Atg14L are the two stable components in distinct Beclin 1-VPS34 complexes and regulate VPS34 activity. We thus investigated the effect of beclin 1 deficiency on UVRAG or Atg14L levels and their associated VPS34 activities. In HeLa cells, knock-down of beclin 1 had a striking impact in the UVRAG protein levels, nearly eliminating the entire intracellular pool of UVRAG protein (Figure 3C). Consistently, UVRAG protein was almost undetectable in *Becn1* deficient MEFs, but was brought back to normal levels in *Becn1* revertant MEFs (Figure 3D). Immunoprecipitation with anti-UVRAG antibody pulled down hardly-visible amounts of UVRAG protein in *Becn1* deficient MEFs, compared to wildtype control or revertant MEFs, correlated with a complete loss of UVRAG-associated VPS34 activity (Figure 4E). Interestingly, Atg14L protein level was reduced but clearly detectable in *Becn1* deficient MEFs, and similarly it was recovered in *Becn1* revertant MEFs (Figure 4F). Measurement of Atg14L-associated VPS34 activity showed approximately 65% reduction in *Becn1* deficient MEFs as compared to that of WT control or revertant MEFs (Figure 4F). Our results indicate that loss of beclin 1 has a much more severe impact on UVRAG protein levels as well as UVRAG-associated VPS34 activity, compared that of Atg14L, and causes a complete loss of the UVRAG-VPS34 complex.

### Disruption of autophagy in *Becn1* deficient fibroblasts

**Figure 5:**
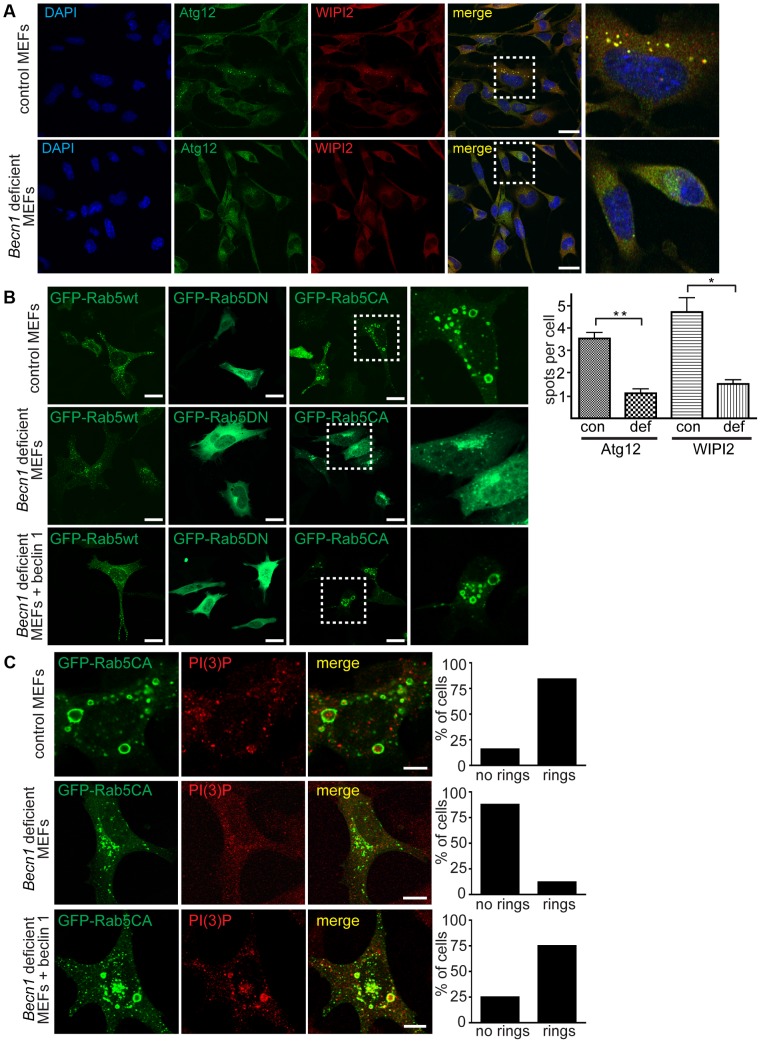
Autophagosome formation and endosomal expansion caused by constitutively active Rab5 and endocytosis are disrupted in *Becn1* deficient MEFs. **A**. Reduction of Atg12 and WIPI2 [56] puncta formation in *Becn1* deficient MEFs. DAPI, endogenous Atg12, WIPI2. White dashed boxes represent zoomed regions. Scalebars = 20 µm. ‘con’ is control MEFs, ‘def’ is beclin 1 deficient MEFs. Quantification of an average of 91 cells per condition from three separate experiments (con: 91, 110, 110; def: 64, 84, 87). Bars represent mean +/− s.e.m. (n = 3); Atg12 p = 0.0039; WIPI2 p = 0.0183 using a one-tailed t-test. **B**. Rab5-CA mutant is mislocalized in *Becn1* deficient MEFs and rescued with re-introduction of beclin 1. MEF cells were transfected with Rab5wt, CA or DN-GFP and visualized. White dashed boxes represent zoomed regions. Scale bars = 20 µm. **C**. PI(3)P is recruited to expanding endosome sites in control MEFs but no endosome expansion associated with Rab5CA-GFP was found in *Becn1* deficient MEFs. MEF cells were transfected with Rab5CA-GFP, fixed, stained with PI(3)P antibody and visualized. Scalebars = 10 µm. Quantification of percentage of cells with no rings or rings (at least 30 transfected cells from at least 3 separate experiments). Control MEFs (7 cells with no rings, 37 cells with rings) vs. *Becn1* deficient MEFs (36,5) significantly different (p<0.0001) and *Becn1* deficient MEFs (36,6) vs. *Becn1* revertant MEFs (11,33) significantly different (p<0.0001) using Fisher's exact test (two-sided).

To further characterize the *Becn1* deficient MEFs we performed several assays to assess autophagy activity in the mutant cells. WIPI2- and Atg12-associated phagophore numbers were significantly reduced in the absence of beclin 1 (Figure 5A). Consistent with the results from studies of Atg6 function in yeast, LC3 lipidation was not inhibited in *Becn1* deficient MEFs in both basal and amino acid starvation conditions (Figure S4A). The number of mature autophagosomes (red spots) as well as immature autophagosomes (yellow spots) as visualized using the mCherry-GFP-LC3 fusion protein reporter were decreased in *Becn1* deficient MEFs (Figure S4B). Also, long-lived protein degradation was inhibited in starvation conditions in *Becn1* deficient MEFs. (Figure S4C). These results are reminiscent of the impairment of autophagy observed in VPS34 deficient cells [27].

### Disruption of active Rab5 GTPase-induced endosome formation in *Becn1* deficient fibroblasts

To further characterize the role of beclin 1 in VPS34-mediated endocytosis, we transfected control and *Becn1* deficient MEFs with plasmids encoding variants of Rab5, a small GTPase that binds EEA1 [28] and the VPS34/Beclin 1 complex [29] and regulates early endosome trafficking [30]. Transfection of GFP-tagged Rab5 wildtype in control MEFs resulted in endosomal localization of GFP-Rab5wt as shown in the green fluorescent puncta. The distribution pattern of both GFP-Rab5 wildtype or dominant negative, GDP-bound mutant Rab5S34N (GFP-Rab5DN) [31] were not obviously changed in *Becn1* deficient MEFs (Figure 5B). However, the constitutively active, GTP-bound mutant Rab5Q79L (GFP-Rab5CA) was frequently associated with enlarged, ring-shaped endocytic vesicles [32] in control MEFs but not in *Becn1* deficient MEFs. Re-introduction of beclin 1 into *Becn1* deficient MEFs by stably expressing wildtype beclin 1 induced a recovery of the enlarged endocytic vesicles. The distinct GFP-Rab5CA vesicles were decorated with PI(3)P, indicating that constitutively active Rab5 leads to excessive recruitment of VPS34 activity [33] and resulting endosome expansion, which was abolished in the absence of beclin 1 (Figure 5C). This result suggests that beclin 1 is required for the Rab5 function in recruiting VPS34 related to endosome function [24].

### Impaired GFP-p40^phox^-associated endosome formation in *Becn1* deficient cells is rescued by overexpression of UVRAG

**Figure 6:**
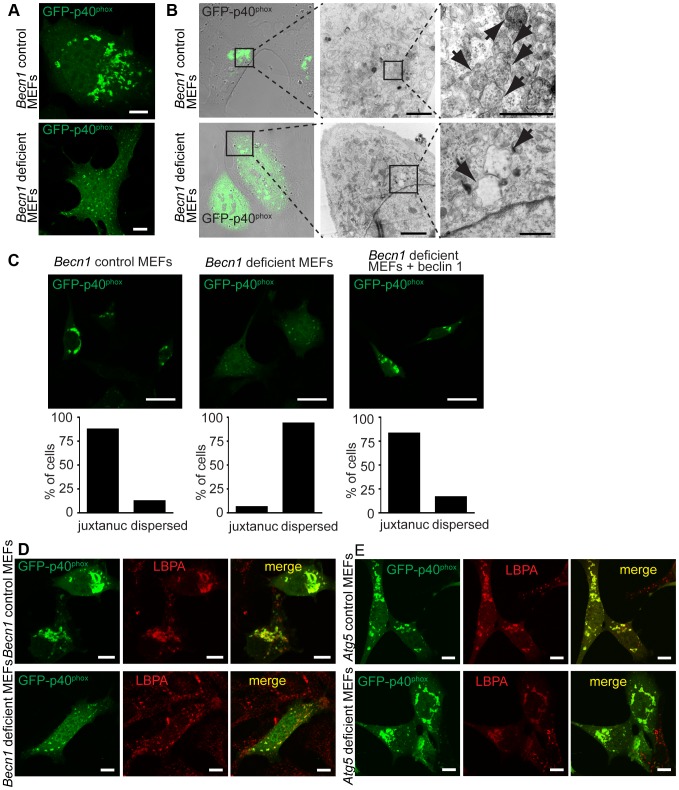
There is a failure of endosome formation and localization in *Becn1* deficient MEFs. **A**. Transfected GFP-p40^phox^ is seen in tight, bright juxta-nuclear structures in control MEFs but is dispersed in *Becn1* deficient MEFs. Images of control and *Becn1* deficient MEFs transfected with GFP-p40^phox^. Scalebars = 10 µm. **B**. GFP-p40^phox^ fluorescence aligns with endosomes in control MEFs but is dispersed in *Becn1* deficient MEFs where enlarged, empty vesicles are observed. Confocal and phase-contrast images and corresponding correlative EM images in control and *Becn1* deficient MEFs transfected with GFP-p40^phox^. Boxes show sequential enlarged areas. Arrows indicate endosomes. Scalebars = 2 µm, 500 nm (LtoR). **C**. GFP-p40^phox^ signal is altered in *Becn1* deficient MEFs. Immunofluorescent images of control, *Becn1* deficient and *Becn1* revertant MEFs that were transfected with GFP-p40^phox^ and fixed. Scalebars = 25 µm. Quantification of percentage of cells with tight juxtanuclear (‘juxtanuc’) or light, dispersed GFP-p40^phox^ staining (at least 30 cells from at least 3 separate experiments). Control MEFs (28,4) vs. *Becn1* deficient MEFs (2,30) significantly different (p<0.0001) and *Becn1* deficient MEFs (2,30) vs. *Becn1* revertant MEFs (25,5) significantly different (p<0.0001) using Fisher's exact test (two-sided). **D**. GFP-p40^phox^ colocalizes with MVB marker LBPA. Immunofluorescent images of control and *Becn1* deficient MEFs that were transfected with GFP-p40^phox^, fixed, and stained with anti-LBPA antibody. Scalebars = 10 µm. **E**. Deletion of autophagy protein Atg5 in MEFs does not affect intracellular GFP-p40^phox^ distribution (does not lead to the GFP-p40^phox^ dispersion phenotype seen in *Becn1* deficient MEFs). GFP-p40^phox^ is still seen in tight, bright juxta-nuclear structures in *Atg5* deficient MEFs as it is in control MEFs. *Atg5* deficient MEFs and corresponding control MEFs were transfected with GFP-p40^phox^, fixed, and stained with anti-LBPA antibody. Scalebars = 10 µm.

To further investigate beclin 1's role in the endocytic pathway we expressed green fluorescent p40^phox^ protein (GFP-p40^phox^), a PI(3)P binding protein and reporter for VPS34 activity [34], in *Becn1* deficient MEFs. Overexpression of GFP-p40^phox^ resulted in intense perinuclear fluorescence in control MEFs but reduced and disperse fluorescence in *Becn1* deficient MEFs (Figure 6A). We performed correlative EM to investigate GFP-p40^phox^ ultrastructures. In control MEFs the reporter protein labeled endosomes that clustered in a perinuclear area (Figure 6B). This result is consistent with previous observations that GFP-p40^phox^ is located on endosomes [35], [36]. However, in the *Becn1* deficient cells the fluorescent GFP-p40^phox^ was not associated with endosomes and the few endosome-like vacuoles detected appear empty (Figure 6B). GFP-p40^phox^-labeled structures are altered in *Becn1* deficient MEFs but restored to bright, juxtanuclear structures in *Becn1* revertant MEFs (Figure 6C). Staining of GFP-p40^phox^ structures with antibody against lipid lysobisphosphatidic acid (LBPA) (Figure 6D) [37] further supports that these structures are late endosomes. Importantly, when the GFP-p40^phox^ was expressed in autophagy gene *Atg5* deficient MEFs the dense, perinuclear GFP-p40^phox^ signal was not altered compared to wildtype MEFs, suggesting the impact on endosome formation is beclin 1-related, rather than Atg5- or autophagy-dependent (Figure 6E).

**Figure 7:**
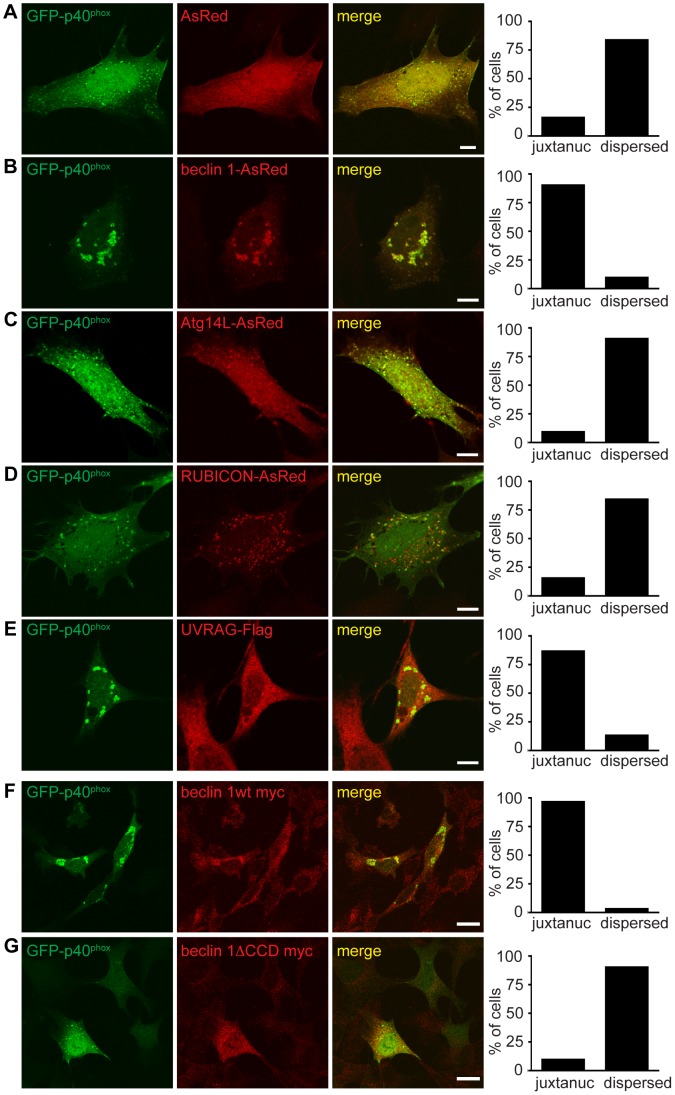
Failure of endosome formation in *Becn1* deficient MEFs is rescued by forced UVRAG expression and requires beclin 1-UVRAG interaction through beclin 1's CC domain. **A–E**. Dispersion of GFP-p40^phox^ in *Becn1* deficient MEFs is rescued with reintroduction of beclin 1 as well as overexpression of UVRAG but not Atg14L or RUBICON. Images of *Becn1* deficient MEFs cotransfected with GFP-p40^phox^ and AsRed (**A**), beclin 1-AsRed (**B**), Atg14L-AsRed (**C**), RUBICON-AsRed (**D**) and UVRAG-Flag and stained with anti-Flag antibody (**E**). Scale bars = 10 µm. Quantification as in Figure 6 E. AsRed (5,26) vs. beclin 1-AsRed (28,3) significantly different (p<0.0001); AsRed (5,26) vs. Atg14L-AsRed (3,29) not significantly different (p = 0.4741); AsRed (5,26) vs. RUBICON-AsRed (5,27) not significantly different (p = 1.0000); AsRed (5,26) vs. UVRAG-Flag (26,4) significantly different (p<0.0001) all using Fisher's exact test (two-sided). Rescue of the aberrant GFP-p40^phox^ distribution in *Becn1* deficient MEFs by beclin 1 overexpression requires the CC domain of beclin 1. A beclin 1 mutant lacking the CC domain does not rescue the GFP-p40^phox^ phenotype of *Becn1* deficient MEFs. Immunofluorescent images of *Becn1* deficient MEFs transfected with beclin 1-myc wt (**F**) or beclin 1-myc minus its UVRAG-binding CC (beclin 1ΔCCD-myc) (**G**) then fixed and stained with anti-myc. Scalebars = 20 µm. Quantification as in Figure 6 E. beclin 1-myc wt (29,1) vs. beclin 1ΔCCD-myc (28,3) significantly different (p<0.0001) using Fisher's exact test (two-sided).

Because beclin 1 forms multiple VPS34 complexes via binding different proteins [8], we next asked if the impairment of endosome formation is related to alteration of specific beclin 1 complexes. We overexpressed beclin 1 or beclin 1-binding proteins in *Becn1* deficient MEFs and examined their effects on GFP-p40^phox^-associated endosome formation. As expected, overexpression of AsRed-tagged beclin 1 reversed the weak, dispersed GFP-p40^phox^ signals to bright perinuclear clusters of GFP-p40^phox^ (Figure 7A,B). We observed a large amount of colocalization between GFP-p40^phox^ and beclin 1-AsRed, confirming our finding that beclin 1-GFP associates with endosome structures in the cerebellar PCs (Figure 2D,E,F). While Atg14L-AsRed or RUBICON-AsRed overexpression did not alter the impaired GFP-p40^phox^ signals (Figure 7 C, D), UVRAG-FLAG overexpression resulted in nearly-complete restoration of GFP-p40^phox^ to a bright, perinuclear cluster pattern, similar to beclin 1 expression (Figure 7E). This result demonstrates that the impairment of GFP-p40^phox^-associated endosome formation is reversed by forced expression of UVRAG in *Becn1* deficient cells, and following on, suggests that the lack of GFP-p40^phox^-associated endosomes in *Becn1* deficient cells is caused by the loss of UVRAG.

### A UVRAG-binding mutant of beclin 1 failed to rescue the impaired GFP-p40^phox^-associated endosomes in *Becn1* deficient cells

Overexpression of beclin 1 mutants able to bind UVRAG but not Atg14L [38] rescued the impaired GFP-p40^phox^ signals in *Becn1* deficient MEFs in a similar manner to beclin 1 wildtype or UVRAG overexpression (Figure S5A). *Becn1* deficient MEFs transfected with a beclin 1 monomer mutant (MutM) or a beclin 1 dimer mutant (MutStab) [38], both of which lose Atg14L binding but retain UVRAG binding, displayed bright juxtanuclear GFP-p40^phox^ signals as seen in control MEFs (Figure 6A) or *Becn1* deficient MEFs transfected with UVRAG (Figure 7E). This is consistent with the notion that Beclin1-Atg14L interaction is dispensable for proper late endosome formation.

Finally, we measured the ability of a UVRAG binding mutant of beclin 1 to rescue irregular GFP-p40^phox^ distribution in *Becn1* deficient MEFs. Beclin 1wt-myc transfection rescued abnormal GFP-p40^phox^ distribution in *Becn1* deficient MEFs just as beclin 1-AsRed did (Figure 7F,G). However, overexpression of beclin 1 missing its coiled-coil domain (beclin 1ΔCCD-myc) failed to rescue the GFP-p40^phox^ dispersion phenotype in *Becn1* deficient MEFs. Given that the beclin 1ΔCCD-myc mutant fails to bind UVRAG, this result suggests that the interaction of beclin 1 with UVRAG via their coiled-coil domains is critical for an intact endosome pathway.

## Discussion

Our study identifies a key role for beclin 1 in the maintenance of homeostatic levels of multiple protein components of the PI3K-III complexes, including UVRAG, Atg14L and the catalytic subunit VPS34 as well as intracellular PI(3)P content. Importantly, beclin 1 is essential for UVRAG-VPS34 subcomplex integrity, which regulates the endosomal pathway. Furthermore, our data shows that lack of beclin 1 causes severe neurodegeneration in mice involving the disruption in both endocytosis and autophagy.

Mounting evidence links beclin 1 to an array of physiological functions and pathological pathways, which were ascribed mostly to its autophagy function [39]. Indeed, a previous report suggested that beclin 1 primarily engages VPS34 in autophagy, refuting the involvement of beclin 1 in VPS34-mediated non-autophagic vesicle trafficking [40]. Our current study demonstrates that beclin 1 participates in multiple organelle trafficking pathways including autophagy and endocytosis in neurons and fibroblasts. Beclin 1 or beclin 1 homologs were previously implicated in the degradative endocytic pathway in *C. elegans* [41], Drosophila [42] and mammalian cells [24] but the mechanisms were unclear.

Our current study suggests that the endocytic functions of beclin 1, strongly tied to UVRAG and VPS34 protein levels as well as VPS34 lipid kinase activity that produces PI(3)P, are constitutive and important for neuron viability, as we observe the collapse of endocytic trafficking associated with aberrant PI(3)P distribution and reduced levels due to loss of beclin 1. Beclin 1 null neurons display severe and accelerated degeneration as compared to autophagy-specific Atg5 or Atg7 deficient mice [43], [44], most likely as a result of combined deleterious effect of deficient autophagy and endocytosis. Our study therefore provides genetic proof for a previously poorly understood function of beclin 1 and expands our knowledge regarding various beclin 1-associated pathogenic mechanisms including those of tumorigenesis and neurodegenerative diseases.

Our study demonstrates a role for beclin 1 in proper endosome formation or maturation and PI3K-III-mediated endocytosis that is functionally separate from its role in autophagy in neurons and fibroblasts. Consistently, upon examination of EM images of both 3T3 cells after knock-down with beclin 1 siRNAs, we found an increase of large, empty vacuoles (Figure S5B) and a similar phenomenon is seen in *Becn1* deficient MEFs (Figure S5C). These structures are similar to structures seen in LAMP1-labeled vacuoles in *Becn1* null PCs (Figure 2 J), VPS34 null cells [27], cells treated with a PIKfyve lipid kinase inhibitor that disrupts endomembrane transport [45] and in bec-1 null *C. elegans* [41]. These structures, consisting of perhaps enlarged, aberrant late endosomes, suggest a defect in maturing endocytic compartments.

Our immuno-EM analysis reveals for the first time that beclin 1 localizes to endosomes (including multivesicular endosomes) in neurons under normal conditions (Figure 2D,E,F). The absence of beclin 1 causes impaired recruitment of endosome markers EEA1 and aberrant enlargement of late endocytic vacuoles (LAMP1-positive). In addition, we observed the disruption of active Rab5 GTPase-induced endocytic vesicles in fibroblasts, which we hypothesize may be due to the inability of Rab5 to recruit VPS34 in the absence of beclin 1 [33]. This result resembles the phenotype of loss of VPS34 due to failure of Rab5 to recruit VPS34 activity, which leads to reduced degradation of EGFR mediated by the endocytic pathway [24], [27]. Therefore, the role of beclin 1/Atg6 in PI3K-III-mediated endocytosis seems to be evolutionarily conserved [1]. Nonetheless, despite the present evidence for beclin 1 function in endosomal trafficking, we cannot exclude a possibility that beclin 1 is also involved in autophagosome-endosomes fusion (to form amphisomes), a process that is currently poorly understood. Overexpression of GFP-p40^phox^ leads to bright, juxtanuclear signals that correspond to areas crowded with endosomes (visualized by EM), but in *Becn1* deficient MEFs we observe very few and aberrant endosomes (Figure 6B). In addition to an inhibition of endosome formation we see mislocalization of the GFP-p40^phox^ signal in *Becn1* deficient MEFs. Although the nature of the abnormal GFP-p40^phox^ structures are unclear, it may also suggest that beclin 1 is required to localize the VPS34 kinase complex to the correct membrane formation sites.

As expected, disruption of beclin 1 in fibroblasts results in impaired autophagy, shown through multiple autophagy assays. Phagophore numbers, as indicated by WIPI2- and Atg12-positive spots were greatly diminished in *Becn1* deficient MEFs (Figure 5A), which may be explained by a decrease in PI(3)P formation in the absence of beclin 1 (Figure 4). This is consistent with the role of WIPI2 as a linker of PI(3)P to the Atg12-5-16L and LC3 conjugation systems at the site of autophagosome formation [46]. It is worth mentioning that, unlike Atg genes that are involved in these ubiquitin-like conjugation systems, lack of beclin 1 does not abolish lipidated LC3II production; instead, it causes unchanged or even elevated levels of LC3II in fibroblasts (Figure S4B) and neurons (Figure S3A), consistent with the requirement of beclin 1 for LC3II-containing autophagosome degradation by lysosomes. Interestingly, we noted that siRNA knock-down of Beclin 1 in HeLa cells and genetic disruption of beclin 1 in MEFs altered the ratio of LC3II to LC3I under normal culture conditions (Figure 3C, Figure S4B), but *Becn1* deficient MEFs showed little change in autophagy flux after bafilomycin A treatment (Figure S4B). While the cause of these observations remains to be tested, we speculate that a compensatory mechanism may be activated to degrade LC3II in the absence of beclin 1, such as beclin 1 homolog beclin 2-mediated autophagy [47]. Overall, autophagy was inhibited in starvation conditions in *Becn1* deficient MEFs as measured by long-lived protein degradation (Figure S4C). It may be suggested that in the absence of beclin 1, the VPS34 kinase complex cannot be targeted to its appropriate membrane, whether that membrane be the phagophore that leads to autophagosomes or endosomal formation sites that become endosomes. This in turn, may lead to unregulated phosphorylation of PI(3)P, which combined with an overall reduction of PI(3)P production in the absence of beclin 1 (Figure 4) causes disruption of multiple membrane trafficking pathways such as autophagy and endocytosis.

Finally, our data suggests that the function of beclin 1 in endosome pathways depends on the interaction between beclin 1 and UVRAG, but not Atg14L or RUBICON, all of which are important binding partners of beclin 1 that exist in distinct beclin 1 complexes and modulate VPS34 lipid kinase activity. Thus our study suggests that the function of the beclin 1-UVRAG complex in the endosome pathway is distinct from its autophagy function primarily associated with the beclin 1-Atg14L complex. In fact, it was previously shown that UVRAG promotes endocytic trafficking [48], [49], [50] and interestingly, lack of beclin 1 results in a complete loss of the UVRAG-VPS34 complex (and associated kinase activity) as opposed to only a partial reduction of Atg14L-VPS34 complex levels (and associated kinase activity) (Figure 7A,B). Therefore, instability or loss of UVRAG in beclin 1 deficient cells is likely to account for the defective endocytosis, which can be rescued by forced expression of UVRAG. In addition, our previous data suggests that the coiled-coil domain interaction between beclin 1 and UVRAG has higher affinity than that of beclin 1 and Atg14L [38]. Therefore, the beclin 1-UVRAG interaction causes a highly stable beclin 1-UVRAG-VPS34 complex that is important for constitutive membrane trafficking (e.g. endocytosis) under normal conditions. Beclin 1 may switch to the Atg14L-containing VPS34 complex when cells face environmental changes that induce autophagy. Adding complexity, however, we cannot exclude the possibility that Atg14L is partially involved in endocytic abnormality in beclin 1 deficient cells [51] due to reduced Atg14L levels.

In summary, our investigation shows that beclin 1 participates in multiple organelle traffic pathways including autophagy and endocytosis via the maintenance of PI3K-III complex integrity and optimal lipid kinase activity and that disruption of multiple PI3K-III-mediated membrane trafficking pathways contributes to neurodegeneration in beclin 1 KO brains. Our data demonstrates a role for beclin 1 in regulating the endosome pathway via its interaction with UVRAG; this role is functionally distinct from autophagy. Our study expands our view of the biological function of beclin 1-VPS34 complex in membrane traffic and improves our understanding of the link between beclin 1 and the pathogenesis of multiple human diseases.

## Materials and Methods

All animal studies were conducted in compliance with the IACUC committee of Icahn School of Medicine at Mount Sinai.

### Generation of beclin 1 Flox/Flox mice

The *Becn1* ^flox/flox^ mice were generated using bacterial artificial chromosome (BAC) recombination techniques in a C57BL/6J strain background. The targeting BAC vector was generated so that two loxP sequences were inserted into the genome to flank the second exon of the *Becn1* gene, where the start codon is located and the neomycin resistant gene pGKneo cassette flanked by two FRT sequences were inserted into the intron region between the second and the third exons (Figure S1A). Purified BAC DNA was electroporated into embryonic stem (ES) cells derived from C57BL/6J-Tyrc-2J/J mice, which are in a C57BL/6J genetic background but have white coat color due to a genetic mutation. Successful germline transmission from a 30% chimera mouse was confirmed with PCR and southern blot. The mice were further crossed to flipper mice to remove the pGKneo cassette flanked by FRT sequences. The resulted *Becn1* ^flox/+^ mice were crossed to each other to generate *Becn1* ^flox/flox^ mice and genotyping was performed using both polymerase chain reaction (PCR) and southern blot (Figure S1 B).

Two shuttle vectors were used to generate the final vector for bacterial artificial chromosome (BAC) modification. S385 contains the two loxP sites and the pGKneo cassette flanked by two FRT sites whereas S351 contains the diphtheria toxin A-fragment gene (DT-A) for negative selection at gene targeting against random integration. A 1000 base pair DNA fragment (box A1) upstream of start codon of *Becn1* gene was PCR amplified using BAC DNA as template and inserted into AscI and EcoRV sites of S351. Another DNA fragment about 500 bp (box A2) immediately downstream of box A1 that contains the second exon of *Becn1* gene was PCR amplified and inserted into EcoRV and KpnI sites of S385. A third DNA fragment about 2000 bp (box B) immediately downstream of box A2 was subcloned into FseI and SmaI sites of S351 and was later used as short arm for homologous recombination in gene targeting. Finally, a 2000 bp DNA fragment (box C) downstream of box B was subcloned into XbaI site of S351, and was used for homologous recombination in the process of BAC modification. Eventually S385 containing all inserted fragments were linearized with NotI and PmeI digestion and ligated into NotI and SwaI sites of S351, generating the complete shuttle vector for BAC modification. Through modification, the loxP-A2-FRT-pGKneo-FRT-loxP fragment was replaced into the BAC clone, and the BAC DNA was used for gene targeting after PI-SceI digestion.

Primers used for cloning “boxes”:

A1 forward: 5′-TCAGGCGCGCCATGTGGGTGGATGGATTTGA-3′, AscI

A1 reverse: 5′-CTACCCGCTGCCTTGGTGGTGGAGG-3′

A2 forward: 5′-CCTCGGGCCCGAGGTTCAAAGGAGC-3′

A2 reverse: 5′-TCAGGTACCTGTAAGAGGATGCACCATGGCAC-3′, KpnI

B forward: 5′-TCAGGCCGGCCGACGAGTTGAGGTTAGAGCCATCAGTG-3′, FseI

B reverse: 5′-TCACCTCATTACCAGCCCCAACCCTACC-3′

C forward: 5′-GAGGCAGGCAGCTGAAGCCAGTGTG-3′

C reverse: 5′-TCAGTTTAAACACCTGCCGAGCCACCCCGTAACA-3′, XbaI

### Gene targeting and blastocyst injection and generation of beclin 1 conditional knock-out mice

Linearized targeting vector was transfected into embryonic stem (ES) cells derived from C57BL/6J mice with electroporation. After that 50 clones were picked and the genomic DNA was tested with southern blot using a 500 bp DNA fragment downstream of box B (short arm of the targeting vector) as the probe. Positive clones were used for blastocyst injection in C57BL/6J^C-2J^ mice. Chimera mice from blastocyst injection were crossed to wild type C57BL/6J mice, and pups with white coat color were genotyped with southern blot using the same probe as above.

For future genotyping analysis, the following primers were used for PCR genotyping:

Forward: 5′-CCACCACCAAGGCAGCGGGTAG-3′

Reverse: 5′-TCACTGATGGCTCTAACCTCAACTCGTC-3′

Positive mice were first crossed to transgenic mice that express FLP recombinase under the control of a broad promoter ROSA26 to remove the PGK-neo cassette flanked by two FRT sites from the genome, and the deletion was confirmed with southern blot.

For genotyping FLP mice, the following primers were used:

Forward: 5′-CTAATGTTGTGGGAAATTGGAGC-3′

Reverse: 5′-CTCGAGGATAACTTGTTTATTGC-3′

*Becn1* ^flox/+^ mice were first crossed to each other to generate *Becn1* ^flox/flox^ mice, while *Becn1* ^flox/+^ mice were crossed with Pcp2-Cre mice to generate *Becn1* ^flox/+;Cre/+^ mice. *Becn1* ^flox/flox^ mice were further crossed with *Becn1* ^flox/+;Cre/+^ mice to generate *Becn1* ^flox/flox;Cre/+^ mice, in which beclin 1 expression is specifically deleted in cerebellar Purkinje cells (Pcp2-Cre) [16].

For genotyping Cre mice, the following primers were used:

Forward: 5′-CCGGTGAACGTGCAAAACAGGCTCTA-3′

Reverse: 3′-CTTCCAGGGCGCGAGTTGATAGC-3′

### Generating L7-beclin 1-GFP mice

The L7-beclin 1-GFP mice were made by inserting a beclin 1-eGFP cDNA right after the ATG of the L7 gene using the same BAC modification protocol [52].

### Generation of *Becn1* deficient MEF cells

Primary mouse embryonic fibroblasts (MEFs) were generated from day 14 to 17 embryos from *Becn1* ^flox/+^ matings. Early passage MEFs were immortalized using an SV40 large T antigen encoding plasmid. Immortalized MEFs were infected with either adenoviral GFP to generate *Becn1* ^+/+^ or adenoviral Cre-GFP to generate *Becn1* ^−/−^ MEFs. Beclin 1 reconstituted MEFs were generated by infecting *Becn1* ^−/−^ MEFs with LPC retroviral vectors expressing beclin 1 (called “*Becn1* deficient+beclin 1 MEFs” or “*Becn1* revertant MEFs”). *Becn1* ^−/−^ control MEFs and *Becn1* ^−/−^ MEFs were transfected with LPC retroviral vectors alone (called “control MEFs” and “*Becn1* deficient MEFs” respectively).

### *In vivo* immunohistochemistry studies

Mice of different ages were put into anesthesia with intraperitoneal Nembutal injection at a dose of 0.01 ml/g body weight. Perfusion was performed with 4% paraformaldehyde in 0.1M phosphate buffer (pH 7.4) and dissected brains were post-fixed in the same fixative at 4°C for overnight. For floating sections, brains were embedded into 5% low melting agarose gel and 40 mm sections were cut with vibrotome (Leica). For cryosections, brains were subjected to cryo-protection in 30% sucrose at 4°C for overnight, and then were embedded into Neg-50 compound (Richard-Allan Scientific). 20 mm sections were cut with a cryostat (Leica). For paraffin sections, brains were dehydrated in series of ethanol (70%, 80%, 90%, 100%) for 30 minutes ×2, followed by incubation in Xylene for 3 days, and were embedded into wax. 10 mm sections were cut with a microtome (Leica). For immuno-fluorescence staining, sections were permeabilized in blocking buffer (10% goat serum and 0.1% Triton X-100 in PBS) and were incubated in primary antibodies diluted in blocking buffer overnight at 4°C. Anti-PI(3)P is from Echelon. Sections were then washed in PBS and incubated in Alexa Fluor conjugated secondary antibodies (Invitrogen) in PBS for 1 hour at room temperature. After washes in PBS, sections were mounted on slide glasses with fluorescence mounting medium (Vector Laboratories) and examined. For HRP staining with paraffin sections, antigen retrieval was first performed and sections were incubated in 0.5% H_2_O_2_ for 15 minutes at room temperature to block the endogenous peroxidase activity. Blocking and primary antibody incubation was performed in the same way as above. Biotinylated secondary antibody reaction was performed using Vectastain Elite ABC Kit (Vector Laboratories), followed by visualization with diaminobenzidine (DAB) (Sigma). For GST-FYVE immuno-EM experiments, cerebellums incubated with GST-FYVE and probed with anti-GST antibody (GE). Microscopes used were Zeiss 510 (Göttingen, Germany) upright and inverted confocal microscopes; 40× and 63× oil objectives were used.

### Electron microscopy (EM)

For morphology EM on tissue sections, animals were perfusion fixed with 2% paraformaldehyde and 2% glutaraldehyde in 0.1M PB, pH 7.4. 40 mm vibrotome-cut sections were cut with. Blocks were cut with a diamond knife on a Leica UltracutE and ultra-thin (∼70 nm) sections were collected on uncoated 200-mesh grids. Grids were viewed with a TecnaiSpiritBT Transmission Electron Microscope (FEI) at 80 KV and pictures were taken with Gatan 895 ULTRASCAN Digital Camera. For morphology EM on cells, transfected HEK 293T cells were fixed in 2.5% glutaraldehyde (2.5GA) in 0.1M cacodylate buffer, pH 7.4 and processed by routine transmission electron microscopy procedure and embedded in Epon [11].

For immuno-EM, transfected HEK 293T cells were fixed in fresh PLP fixative (4% paraformaldehyde, 0.01M periodate, 0.075M lysine, and 0.075M phosphate buffer/pH 7.4) supplemented with 0.05% glutaraldehyde for one hour. After three washes in PBS, cells were permeabilized with 0.01% saponin in PBS supplemented with 0.1% BSA for 15 minutes at room temperature, and were incubated with primary antibody (anti-GFP) diluted in the same buffer overnight at 4°C. Vectastain Elite ABC kit was used for secondary antibody incubation, and cells were fixed again in 1.5% glutaraldehyde in 0.1 M cacodylate buffer supplemented with 5% sucrose. After three washes with 50 mM Tris/pH 7.4 supplemented with 7.5% sucrose, DAB reaction was performed with the same kit. The reaction was stopped with 50 mM Tris/pH 7.4 supplemented with 7.5% sucrose, and cells were processed for silver enhancement as [53]. Blocks were cut with a diamond knife on a Leica UltracutE and ultra-thin (∼70 nm) sections were collected on uncoated 200-mesh grids and stained with Uranium and Lead. Grids were viewed with a TecnaiSpiritBT Transmission Electron Microscope (FEI) at 80 KV and pictures were taken with Gatan 895 ULTRASCAN Digital Camera. Image levels were processed in Adobe Photoshop (Adobe Systems, San Jose, CA) to enhance contrast.

For correlative light and electron microscopy, cells were plated on 35 mm live imaging dishes with gridded glass bottoms (Mattek) for analysis by CLEM. Cells were fixed 24 h after transfection with 4% gluteraldehyde in 0.1 M sodium cacodylate buffer with 1 mM CaCl2. Confocal image stacks of transfected cells were acquired on a Zeiss LSM 510 META microscope. Differential interference contrast (DIC) and fluorescent images were acquired simultaneously. Initially, brightfield and DIC images taken at 10× low magnification were used to document the location of cells with respect to the coverslip grid. This step allows for identification of the cells of interest after processing for TEM. DIC and GFP LSCM z-stack images were then taken using a 63× objective. After imaging was complete, the sample was washed in sodium cacodylate buffer and treated with 1% osmium tetroxide, 1.5% potassium ferracyanide in 0.1 M cacodylate buffer for 1 h at 4°C. Cells were then dehydrated in ethanol using increasing steps of 50%, 60% and 70%, left in 3% uranyl acetate in 70% ethanol overnight, and then dehydrated further in 80%, 90% and 100% ethanol. After dehydration, cells were infiltrated with a 1∶1 mixture of resin (Embed 812 26 kit; Electron Microscopy Sciences) and 100% ethanol for 24 h. Resin/ethanol was replaced with a layer of pure resin, the cells of interest were enclosed by an open ended embedding capsule, then hardened in a vacuum oven at 65°C for 24 h. The capsule was topped off with resin and hardened in the oven for another 24 h. Lastly, the block was separated from the glass bottom dish by being placed on a hot plate at 60°C for ∼2–3 min and carefully peeled free from the dish. The imaged cells of interest were relocated on the resin block face using the superimposed coverslip grid. The block was trimmed using a Diatome cryotrim 45° trimming knife (Electron Microscopy Sciences), then serial sectioned at 70 nm using a Reichardt Ultracut E ultramicrotome. Serial section ribbons were transferred to 2×1 mm carbon reinforced slot grids (Electron Microscopy Sciences) then stained with 3% uranyl acetate for 40 min, washed, then stained with Reynold's lead citrate for 3 min. Cells of interest are initially located at low magnification in order to observed cell morphology and locate specific regions of interest based on comparison to the confocal GFP image. Cells and organelles were then imaged in serial sections at 10,000–30,000× magnification.

### Cell cultures, transfections and constructs

Control MEFs, *Becn1* deficient MEFs, *Becn1* deficient plus beclin 1 MEFs, Human Embyronic Kidney (HEK) 293T cells, HeLa, and NIH 3T3 cells were maintained in Dulbecco's modified Eagle's medium (DMEM) supplemented with 10% fetal bovine serum (Invitrogen, Carlsbad, CA). Transient DNA transfection was performed with either the standard calcium phosphate precipitation procedure, FuGene 6 or Lipofectamine2000, following protocols provided by the manufacturers. The Myc-tagged beclin 1 CCD deleted mutant, lacking the coiled-coil domain (aa 174–266), was constructed by overlapping extension PCR-based deletion mutagenesis and cloned into a PCMV-Myc vector with EcoRI and XhoI restriction sites.

### Immunohistochemistry studies on fixed cells

Cells were fixed with 4% Pfa, permeabilized with 0.2% Triton, blocked in 1% BSA and were stained with anti-PI(3)P (Echelon), LBPA (Echelon), Atg12 (Cell Signaling #2011), WIPI2 (Abcam), Flag-tag M2 (Sigma), and Myc-tag 9B11 (Cell Signaling) and imaged on upright and inverted confocal microscopes made by Zeiss (Göttingen, Germany) including Zeiss 510 and Zeiss 780. 40× and 63× oil objectives were used. For quantification, at least 30 transfected cells imaged from at least three separate experiments were examined and categorized as indicated (i.e. no rings vs. rings).

### EGFR degradation assay

Control, *Becn1* deficient and *Becn1* deficient plus beclin 1 MEFs were plated on poly-d-lysine coated 12 well plates. Once adherent, cells were serum-starved overnight. Cells were then stimulated with 200 ng/mL EGF (Millipore) in serum-free medium for the indicated time points. Cells were lysed in 1% TNTE buffer and homogenized with a 26G needle. Western blots were probed with anti-EGFR (Millipore), anti-beclin 1 (Santa Cruz), anti-UVRAG (Abgent), and anti-actin (Cell Signaling). EGFR/actin was quantified using Metamorph.

### Endocytosis dye flow cytometry assay

Hela cells were treated with siControl or siBeclin siRNA and treated with DMSO or 100 nM Bafilomycin A1 (EMD Chemicals) for 1 hour. Cells were incubated with 50 µg/mL pHrodo Red dextran (MW, 10,000; P10361) or 1 µM LysoSensor Green (L7535) (both life technologies) for 20 minutes. Cells were harvested by trypsination, resuspended in ice-cold 0.2% BSA in PBS and analyzed immediately without fixation by flow cytometry using LSR-II (BD Biosciences) at an excitation/emission ratio of 560/585 nm for pHrodo Red dextran and 443/505 for LysoSensor Green. Mean PE-A fluorescence intensity and mean Am Cyan-A fluorescence intensity for 10,000 cells were determined.

### PI(3)P ELISA

Lipid were extracted from MEF cells and samples were brought up in appropriate sample buffer volume based on lipid pellet weight. The PI(3)P ELISA was performed according to manufacturer's instructions using a PI(3)P Mass ELISA Kit (K-3300, Echelon). 450 nm absorbance was read on a VICTOR X4 plate reader (PerkinElmer) and PI(3)P concentration was determined using a standard curve generated during the ELISA.

### Lipid kinase assay

MEF cells were lysed in 10 mM Tris-HCl pH 7.5, 2 mM EDTA, 100 mM NaCl, 1% NP-40, protease inhibitor cocktail (Roche) and phosphatase inhibitor cocktail (Roche). Immunoprecipitation was performed with 500 µg protein from post-nuclear supernatant, using antibodies against VPS34 (Echelon), UVRAG (Bethyl), or ATG14 (MBL) overnight. Samples were incubated with 30 µl Dynabeads (Life Technologies) for 1–2 hours at 4°C and then washed three times with lysis buffer. Two thirds of the beads were reserved for western blotting, while the remaining one third was used for the kinase assay. Beads for the kinase assay were washed once with wash buffer (20 mM HEPES pH 7.4, 1 mM EGTA, 0.4 mM EDTA, 5 mM MgCl2, and 0.05 mM DTT) and then resuspended to a final volume of 50 µl with reaction buffer (20 mM HEPES pH 7.4, 1 mM EGTA, 0.4 mM EDTA, 5 mM MgCl2, and 0.05 mM DTT, 50 µM cold ATP, 5 mM MnCl2, 0.1 mg/ml sonicated phosphatidylinositol). Note that 10 nm wortmannin was included in the indicated control MEFs. The reaction was started with the addition of γ-32P-ATP (5 µCi) and the samples were shaken at 37°C for 30 minutes. Reactions were stopped with 120 µl stop buffer (CHCl3/CH3OH/HCl at a 10∶20∶0.2 volume ratio) and then shaken for another 10 minutes at room temperature. Samples were centrifuged for 5 minutes at 1000 rcf. 15 µl of the lower, organic phase was resolved on silica coated TLC plate (Millipore) using CHCL3/CH3OH/NH4OH/H2O (86∶76∶10∶14 volume ratio) and visualized with the Typhoon 9400 Variable Imager (GE Healthcare Biosciences).

### Autophagy assays and long-lived protein degradation

Autophagy assays were performed as in [6] and [54]. Long-lived protein degradation was assessed as described previously [55]. Control and *Becn1* deficient MEFs were plated in 12-well plates. After 48 h, DMEM was changed to leucine-free medium supplemented with 3H-l-leucine (1 µCi ml–1). After pulse-labeling for 24 h, cells were washed three times and cultured in DMEM supplemented with excess unlabelled leucine (5 mM) for 16 h to chase out short-lived proteins. Cells were then washed three times and further cultured for 4 h in DMEM, Earle's Balanced salt solution (EBSS), or EBSS supplemented with 10 mM 3-methyladenine, all containing unlabelled leucine (5 mM). Both medium and cell lysates were subject to trichloroacetic acid (TCA) precipitation. Long-lived protein degradation was calculated as the ratio of TCA- soluble medium to TCA-precipitated cell lysate radioactivity.

## Supporting Information

Figure S1 Generation of *Becn1* ^F/F-Pcp2-Cre^ mice to delete *Becn1* specifically in Purkinje cells (PCs). **A**. Schematic for the generation of mice carrying floxed *Becn1* alleles (*Becn1* ^F/F^), used to cross with mice expressing Cre recombinase to create conditional KO mice. **B**. Top: DNA gel; bottom: southern blot, showing *Becn1* wild-type (+/+), (+/Flox), and (Flox/Flox) genotypes. **C**. Beclin 1 protein is successfully depleted in Purkinje cells of *Becn1* cerebellum cKO mice. Cerebellum sections from *Becn1* ^F/F^ and *Becn1* ^F/F-Pcp2-Cre^ mice at the age of P21 were stained with anti-beclin 1 antibody. Lower panels show zoom of boxed region. The dashed boxes highlight Purkinje cell bodies. Scale bar = 200 µm. **D**. PCs are mostly intact at P21, suggesting that loss of beclin 1 staining is not due to PC degeneration. Calbindin 1 staining of cerebellums from *Becn1* ^F/F^ and *Becn1* ^F/F-Pcp2-Cre^ mice at P21. Lower panels show zoom of boxed regions. Scalebar = 200 µm.
(TIF)

Figure S2 Loss of beclin 1 in the cerebellum leads to locomotor deficiency and abnormal axon morphology. **A**. Loss of beclin 1 in the cerebellum PCs leads to locomotor abnormality. 1-month-old (1M) *Becn1* ^F/F;Pcp2-Cre^ mice show abnormal gait patterns. Image after the front paws were painted red and hind paws painted green and the mice trained to walk through a tunnel, stamping paper as they moved forward. **B**. cKO of *Becn1* in the hippocampus/cortex leads to reduced body size. *Becn1* ^F/F;EMX-Cre^ hippocampus/cortex-specific KO mice show reduced size. Photos of *Becn1* ^F/F^ and *Becn1* ^F/F;EMX-Cre^ mice at 1.5M, 2M. **C**. Purkinje cell axon termini are abnormal in *Becn1* ^F/F;Pcp2-Cre^ mice. EM ultrastructural analysis of Purkinje cell axon terminus in the deep cerebellar nuclei (DCN) area at P28. Swollen, abnormal myelinated PC axons (arrowhead) in *Becn1* ^F/F;Pcp2-Cre^ mice contain accumulations of electron-dense membranous structures that are undigested vesicles and membranes. Scale bars = 100 µm.
(TIF)

Figure S3 Autophagy is impaired in the *Becn1* deficient hippocampal neurons. **A**. p62 and LC3 protein levels increase after *Becn1* loss in the hippocampus CA1. Anti-p62, beclin 1, LC3 and actin blots in hippocampal brain lysates of *Becn1* ^F/F^ or *Becn1* ^F/F;EMX-Cre^ mice at P12. **B**. Increased p62 aggregate formation is observed in *Becn1* ^F/F;EMX-Cre^ brains, which are found in cell bodies of hippocampal cells. Hippocampal slices from P12 and P15 *Becn1* ^F/F;EMX-Cre^ mice were stained with NeuN and p62. Middle panels show zoomed images of top panels. Scale bars = 100 µm, 10 µm, 100 µm respectively. **C**. p62 accumulation and an increase in GFP-LC3 puncta are observed in beclin 1 knock-out hippocampi from P12 *Becn1* ^F/F^-GFP-LC3 control versus *Becn1* ^F/F;EMX-Cre^-GFP-LC3 brain slices stained for p62. Scale bars = 100 µm. Dashed lines show zoomed boxes. **D**. Ubiquitin accumulation is observed in beclin 1 knock-out hippocampi from P12 *Becn1* ^F/F^-GFP-LC3 control versus *Becn1* ^F/F;EMX-Cre^-GFP-LC3 brain slices stained for ubiquitin. Scale bars = 100 µm. Dashed lines show zoomed boxes.
(TIF)

Figure S4 *Becn1* deficient MEFs display decreased autophagy activity. **A**. LC3 lipidation was not grossly affected in both basal and starvation-induced autophagy in *Becn1* deficient MEFs in both the presence and absence of the lysosomal inhibitor bafilomycin. Anti-beclin 1, -actin, and –LC3 blots in control and beclin 1 deficient MEFs treated for 2H with indicated medium. FM is full medium, FB is FM plus 100 nM bafilomycin, ES is Earl's Buffered Salt Solution, EB is ES plus 100 nM bafilomycin. **B**. Overall signals of mCherry-GFP-LC3 fluorescence are reduced in *Becn1* deficient MEFs. The amount of red and green puncta were both decreased. Control or *Becn1* deficient MEFs were transfected with mCherry-GFP-LC3 and fixed. Scalebars = 10 µm **C**. Loss of beclin 1 in *Becn1* deficient MEFs inhibits long-lived protein degradation induced by amino acid starvation. Ratio of ^3^[H] Leucine. Bars represent mean +/− s.e.m. 3-MA is 3-methyladenine p = 0.0336 using a one-tailed t-test.
(TIF)

Figure S5 Rescue of the aberrant GFP-p40^phox^ distribution in *Becn1* deficient MEFs by beclin 1 overexpression is independent of beclin 1-Atg14L binding and loss of beclin 1 leads to enlarged, translucent vesicles most likely due to impairment of endosomal maturation. **A**. Beclin 1 mutants able to bind UVRAG but not Atg14L rescue the GFP-p40^phox^ dispersion phenotype of *Becn1* deficient MEFs. Immunofluorescent images of *Becn1* deficient MEFs transfected with UVRAG-FLAG, As-Red tagged beclin 1 monomer mutant (MutM) or As-Red tagged beclin 1 dimer mutant (MutStab) [38] and fixed. Cells transfected with UVRAG-FLAG were stained with anti-FLAG antibody. Scalebars = 10 µm. **B**. Large, translucent vesicles throughout the cytoplasm are observed after beclin 1 knock-down. EM images of 3T3 cells transfected with control or beclin 1 siRNA. Scalebars = 5 µm. **C**. Similar large, empty vesicles are observed in beclin 1 deficient MEFs. EM images of control or *Becn1* deficient MEFs. N is nucleus. Scalebars = 500 nm.
(TIF)

Movie S1 *Becn1* ^F/F^ control mice display normal locomotive coordination. Video of 5-month-old control mice in cage.
(MOV)

Movie S2 *Becn1* ^F/F;Pcp2-Cre^ cKO mice display abnormal locomotive coordination. Video of 5-month-old Purkinje cell *Becn1* conditional knockout mice in cage.
(MOV)
