# Supplementary figures and images for: Beclin 1 Is Required for Neuron Viability and Regulates Endosome Pathways via the UVRAG-VPS34 Complex
Source: PLoS Genet. 2014 Oct 2;10(10):e1004626. doi: 10.1371/journal.pgen.1004626 (PMC4183436; doi:10.1371/journal.pgen.1004626)

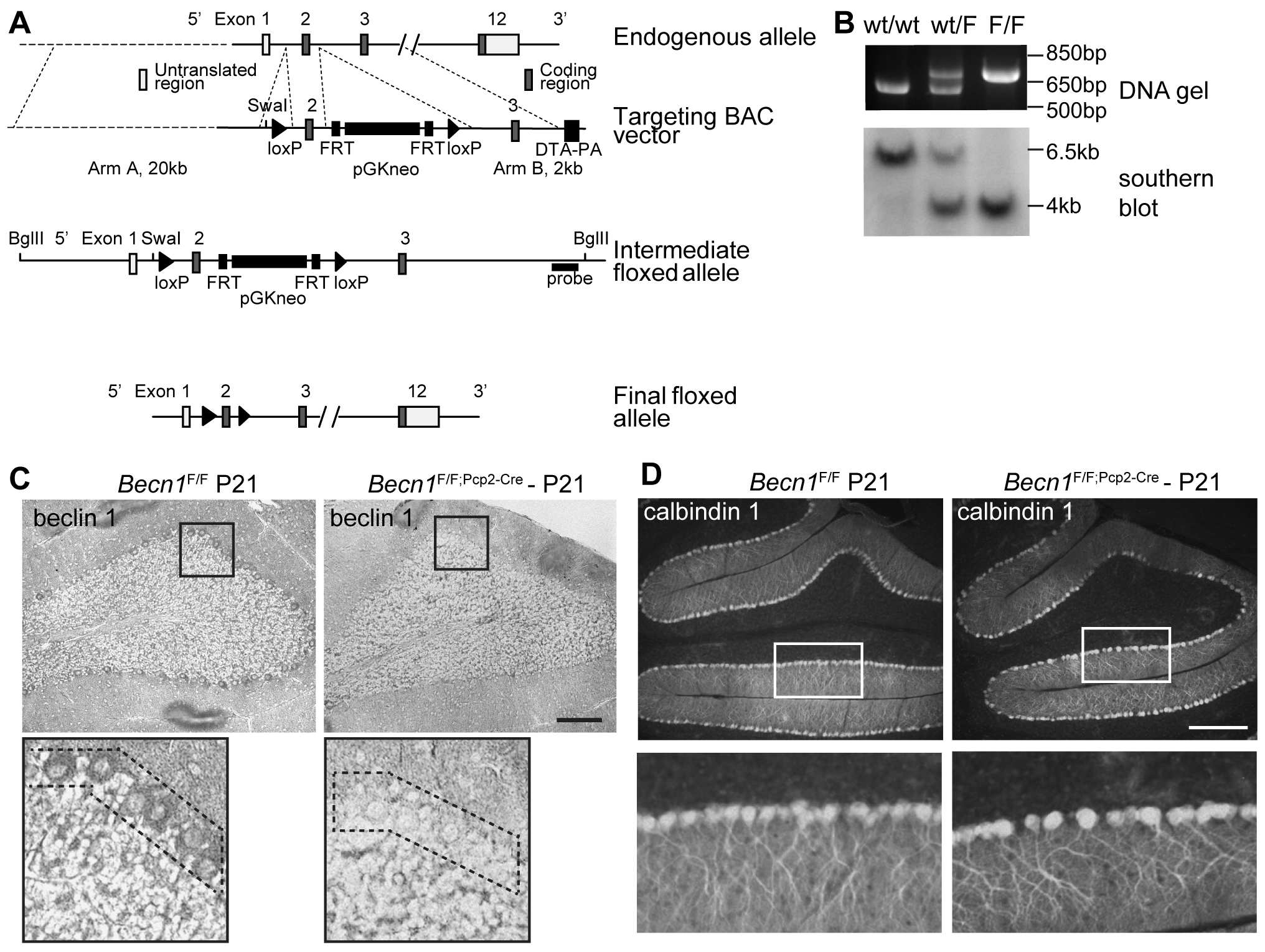

Supplement: Figure S1 — Generation of Becn1 F/F-Pcp2-Cre mice to delete Becn1 specifically in Purkinje cells (PCs). A. Schematic for the generation of mice carrying floxed Becn1 alleles (Becn1 F/F), used to cross with mice expressing Cre recombinase to create conditional KO mice. B. Top: DNA gel; bottom: southern blot, showing Becn1 wild-type (+/+), (+/Flox), and (Flox/Flox) genotypes. C. Beclin 1 protein is successfully depleted in Purkinje cells of Becn1 cerebellum cKO mice. Cerebellum sections from Becn1 F/F and Becn1 F/F-Pcp2-Cre mice at the age of P21 were stained with anti-beclin 1 antibody. Lower panels show zoom of boxed region. The dashed boxes highlight Purkinje cell bodies. Scale bar = 200 µm. D. PCs are mostly intact at P21, suggesting that loss of beclin 1 staining is not due to PC degeneration. Calbindin 1 staining of cerebellums from Becn1 F/F and Becn1 F/F-Pcp2-Cre mice at P21. Lower panels show zoom of boxed regions. Scalebar = 200 µm. (TIF) [file pgen.1004626.s001.tif]

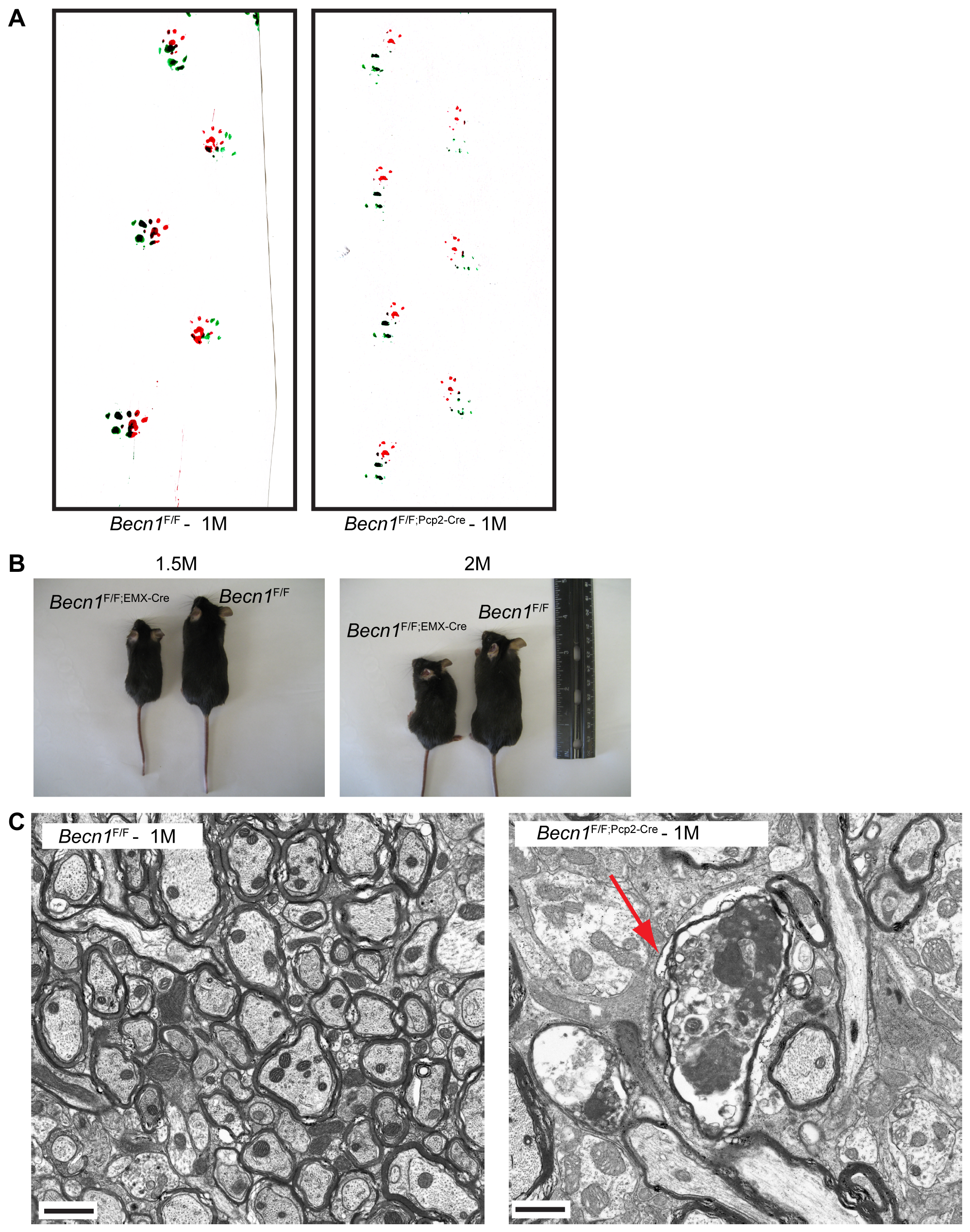

Supplement: Figure S2 — Loss of beclin 1 in the cerebellum leads to locomotor deficiency and abnormal axon morphology. A. Loss of beclin 1 in the cerebellum PCs leads to locomotor abnormality. 1-month-old (1M) Becn1 F/F;Pcp2-Cre mice show abnormal gait patterns. Image after the front paws were painted red and hind paws painted green and the mice trained to walk through a tunnel, stamping paper as they moved forward. B. cKO of Becn1 in the hippocampus/cortex leads to reduced body size. Becn1 F/F;EMX-Cre hippocampus/cortex-specific KO mice show reduced size. Photos of Becn1 F/F and Becn1 F/F;EMX-Cre mice at 1.5M, 2M. C. Purkinje cell axon termini are abnormal in Becn1 F/F;Pcp2-Cre mice. EM ultrastructural analysis of Purkinje cell axon terminus in the deep cerebellar nuclei (DCN) area at P28. Swollen, abnormal myelinated PC axons (arrowhead) in Becn1 F/F;Pcp2-Cre mice contain accumulations of electron-dense membranous structures that are undigested vesicles and membranes. Scale bars = 100 µm. (TIF) [file pgen.1004626.s002.tif]

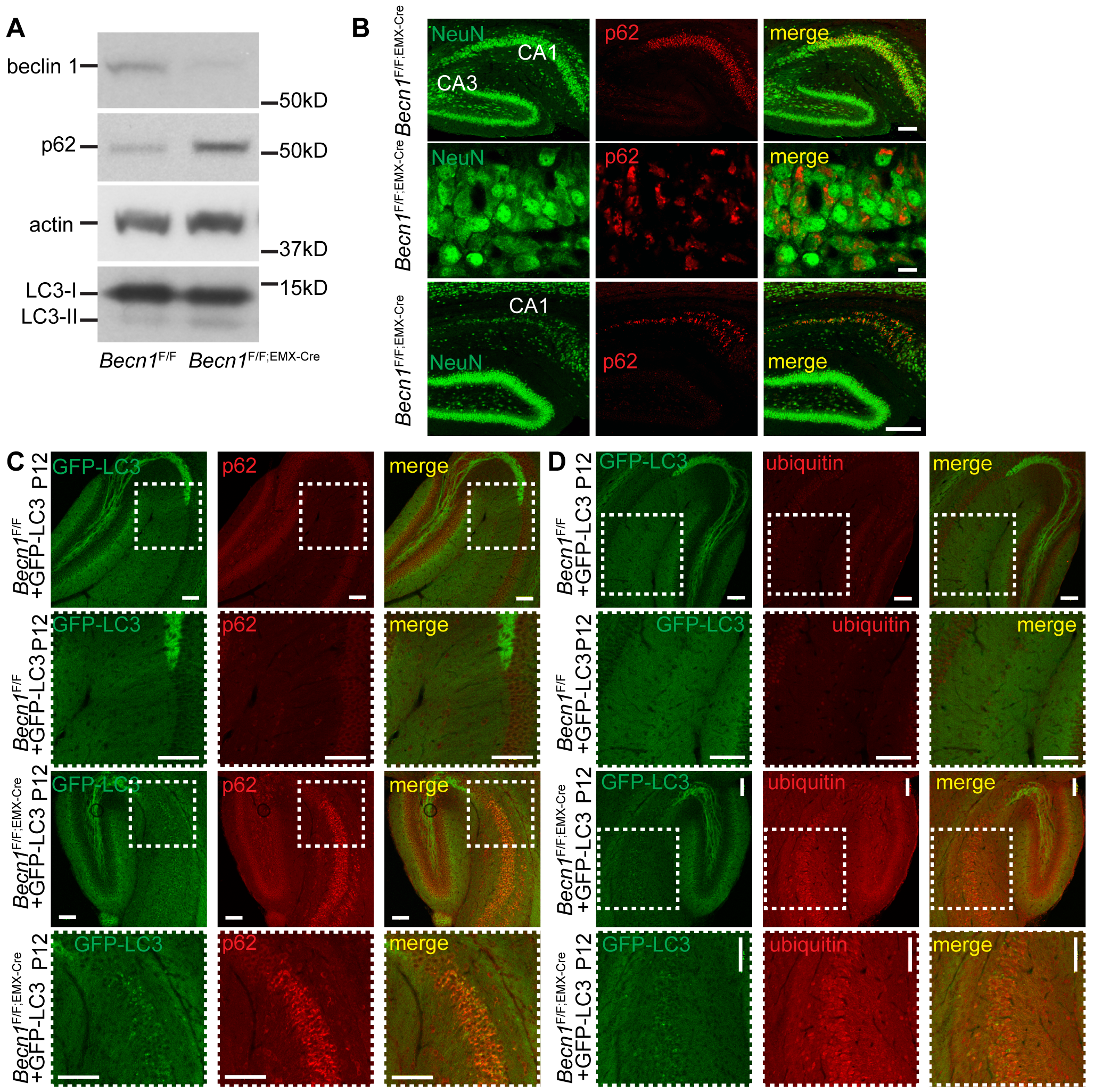

Supplement: Figure S3 — Autophagy is impaired in the Becn1 deficient hippocampal neurons. A. p62 and LC3 protein levels increase after Becn1 loss in the hippocampus CA1. Anti-p62, beclin 1, LC3 and actin blots in hippocampal brain lysates of Becn1 F/F or Becn1 F/F;EMX-Cre mice at P12. B. Increased p62 aggregate formation is observed in Becn1 F/F;EMX-Cre brains, which are found in cell bodies of hippocampal cells. Hippocampal slices from P12 and P15 Becn1 F/F;EMX-Cre mice were stained with NeuN and p62. Middle panels show zoomed images of top panels. Scale bars = 100 µm, 10 µm, 100 µm respectively. C. p62 accumulation and an increase in GFP-LC3 puncta are observed in beclin 1 knock-out hippocampi from P12 Becn1 F/F-GFP-LC3 control versus Becn1 F/F;EMX-Cre-GFP-LC3 brain slices stained for p62. Scale bars = 100 µm. Dashed lines show zoomed boxes. D. Ubiquitin accumulation is observed in beclin 1 knock-out hippocampi from P12 Becn1 F/F-GFP-LC3 control versus Becn1 F/F;EMX-Cre-GFP-LC3 brain slices stained for ubiquitin. Scale bars = 100 µm. Dashed lines show zoomed boxes. (TIF) [file pgen.1004626.s003.tif]

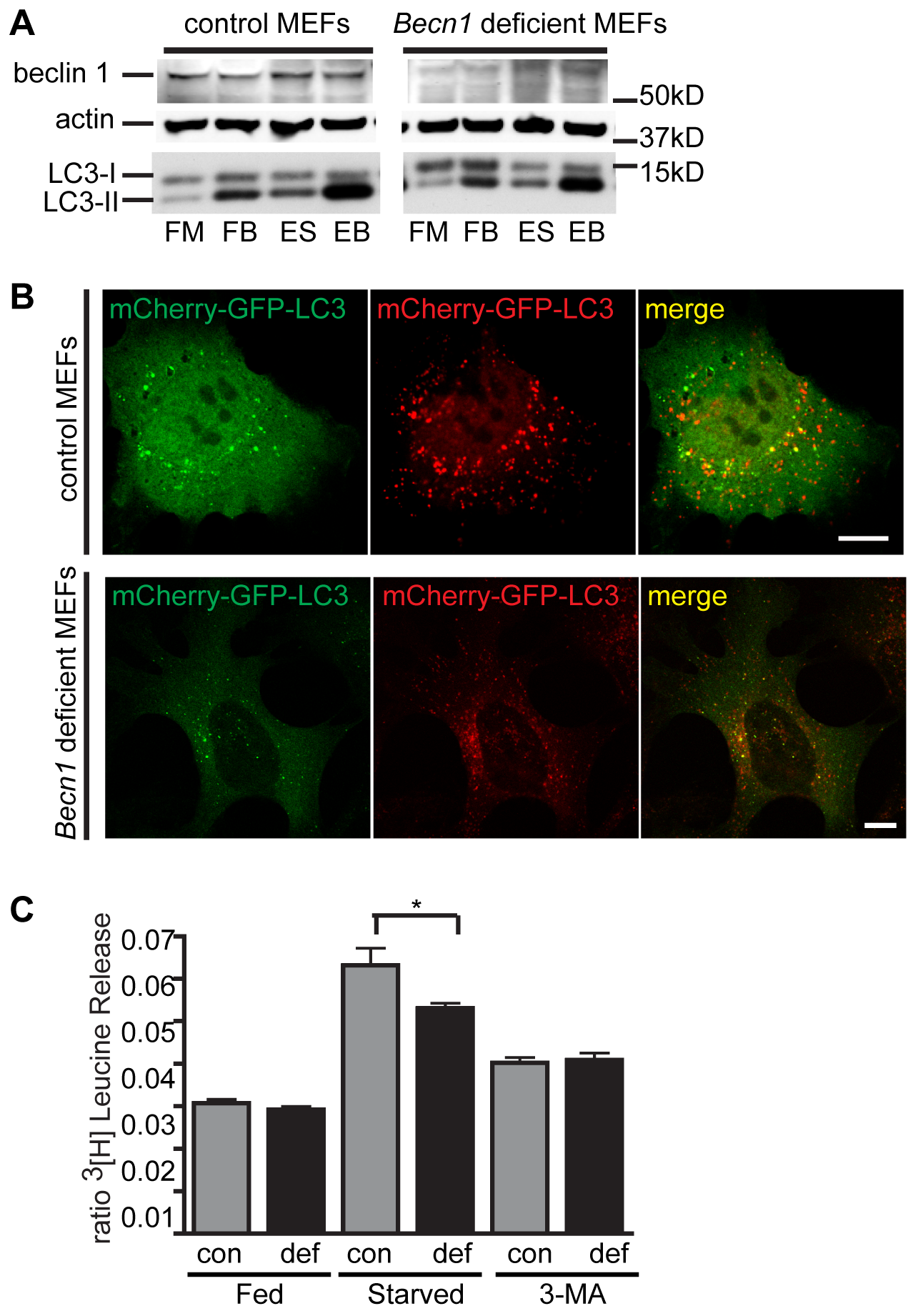

Supplement: Figure S4 — Becn1 deficient MEFs display decreased autophagy activity. A. LC3 lipidation was not grossly affected in both basal and starvation-induced autophagy in Becn1 deficient MEFs in both the presence and absence of the lysosomal inhibitor bafilomycin. Anti-beclin 1, -actin, and –LC3 blots in control and beclin 1 deficient MEFs treated for 2H with indicated medium. FM is full medium, FB is FM plus 100 nM bafilomycin, ES is Earl's Buffered Salt Solution, EB is ES plus 100 nM bafilomycin. B. Overall signals of mCherry-GFP-LC3 fluorescence are reduced in Becn1 deficient MEFs. The amount of red and green puncta were both decreased. Control or Becn1 deficient MEFs were transfected with mCherry-GFP-LC3 and fixed. Scalebars = 10 µm C. Loss of beclin 1 in Becn1 deficient MEFs inhibits long-lived protein degradation induced by amino acid starvation. Ratio of 3[H] Leucine. Bars represent mean +/− s.e.m. 3-MA is 3-methyladenine p = 0.0336 using a one-tailed t-test. (TIF) [file pgen.1004626.s004.tif]

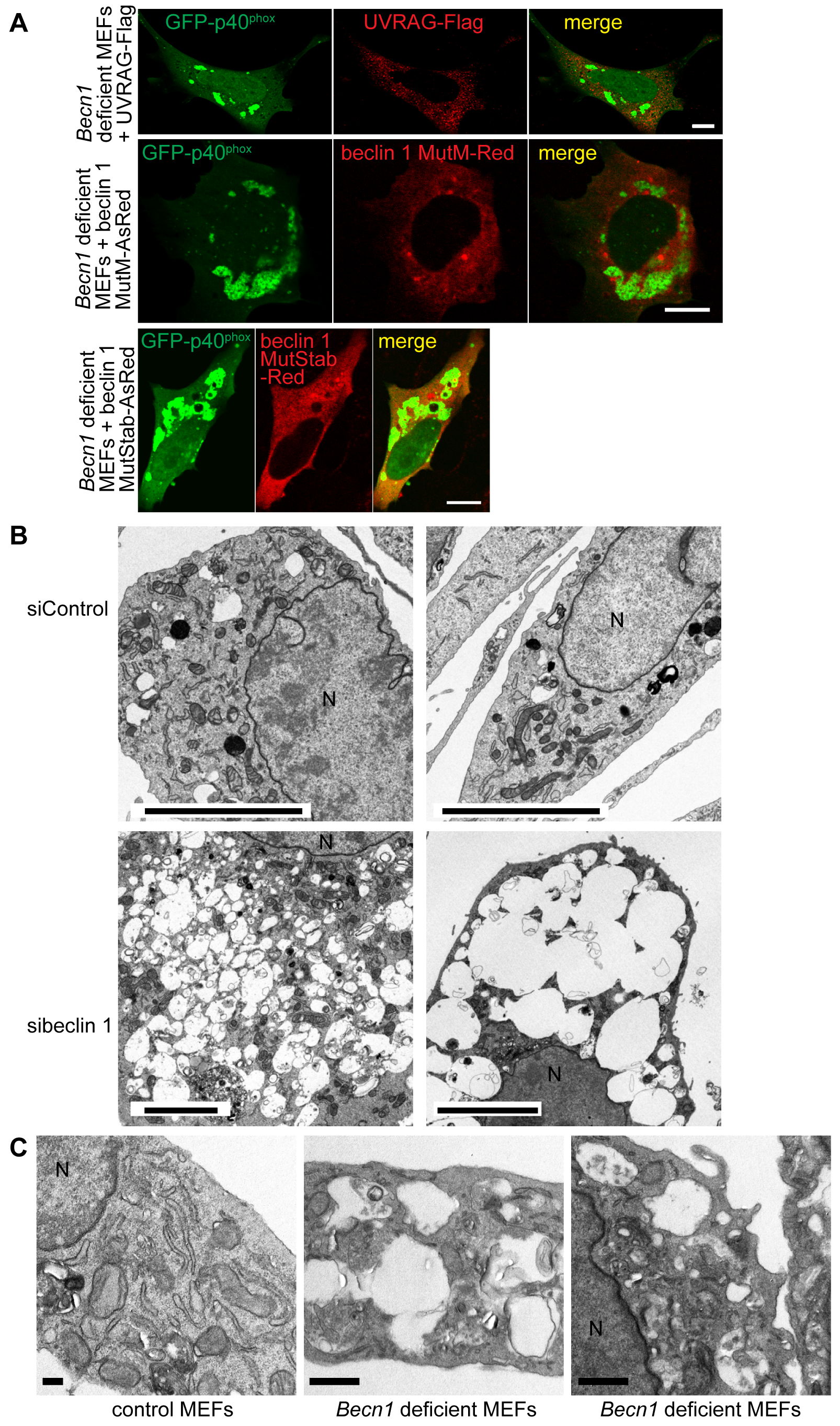

Supplement: Figure S5 — Rescue of the aberrant GFP-p40phox distribution in Becn1 deficient MEFs by beclin 1 overexpression is independent of beclin 1-Atg14L binding and loss of beclin 1 leads to enlarged, translucent vesicles most likely due to impairment of endosomal maturation. A. Beclin 1 mutants able to bind UVRAG but not Atg14L rescue the GFP-p40phox dispersion phenotype of Becn1 deficient MEFs. Immunofluorescent images of Becn1 deficient MEFs transfected with UVRAG-FLAG, As-Red tagged beclin 1 monomer mutant (MutM) or As-Red tagged beclin 1 dimer mutant (MutStab) [38] and fixed. Cells transfected with UVRAG-FLAG were stained with anti-FLAG antibody. Scalebars = 10 µm. B. Large, translucent vesicles throughout the cytoplasm are observed after beclin 1 knock-down. EM images of 3T3 cells transfected with control or beclin 1 siRNA. Scalebars = 5 µm. C. Similar large, empty vesicles are observed in beclin 1 deficient MEFs. EM images of control or Becn1 deficient MEFs. N is nucleus. Scalebars = 500 nm. (TIF) [file pgen.1004626.s005.tif]
